# Genomic analysis of 1710 surveillance-based *Neisseria gonorrhoeae* isolates from the USA in 2019 identifies predominant strain types and chromosomal antimicrobial-resistance determinants

**DOI:** 10.1099/mgen.0.001006

**Published:** 2023-05-12

**Authors:** Jennifer L. Reimche, Arvon A. Clemons, Vasanta L. Chivukula, Sandeep J. Joseph, Matthew W. Schmerer, Cau D. Pham, Karen Schlanger, Sancta B. St Cyr, Ellen N. Kersh, Kim M. Gernert, †Antimicrobial- Resistant Neisseria gonorrhoeae Working Group

**Affiliations:** ^1^​ Division of STD Prevention, National Center for HIV, Viral Hepatitis, STD, and TB Prevention, Centers for Disease Control and Prevention, Atlanta, GA, USA; ^2^​ Oak Ridge Institute for Science and Education Research Participation and Fellowship Program, Oak Ridge, TN, USA; ^3^​ Division of Preparedness and Emerging Infections, National Center for Emerging and Zoonotic Infectious Diseases, Centers for Disease Control and Prevention, Atlanta, GA, USA; ^4^​ Antimicrobial Resistance Coordination and Strategy Unit, Division of Healthcare Quality Promotion, National Center for Emerging and Zoonotic Infectious Diseases, Centers for Disease Control and Prevention, Atlanta, Georgia, USA; ^5^​ Division of HIV Prevention, National Center for HIV, Viral Hepatitis, STD, and TB Prevention, Centers for Disease Control and Prevention, Atlanta, GA, USA

**Keywords:** (3-6) antimicrobial resistance, molecular epidemiology, *Neisseria gonorrhoeae*, surveillance, whole-genome sequencing

## Abstract

This study characterized high-quality whole-genome sequences of a sentinel, surveillance-based collection of 1710 *Neisseria gonorrhoeae* (GC) isolates from 2019 collected in the USA as part of the Gonococcal Isolate Surveillance Project (GISP). It aims to provide a detailed report of strain diversity, phylogenetic relationships and resistance determinant profiles associated with reduced susceptibilities to antibiotics of concern. The 1710 isolates represented 164 multilocus sequence types and 21 predominant phylogenetic clades. Common genomic determinants defined most strains’ phenotypic, reduced susceptibility to current and historic antibiotics (e.g. *bla*_TEM_ plasmid for penicillin, *tetM* plasmid for tetracycline, *gyrA* for ciprofloxacin, 23S rRNA and/or mosaic *mtr* operon for azithromycin, and mosaic *penA* for cefixime and ceftriaxone). The most predominant phylogenetic clade accounted for 21 % of the isolates, included a majority of the isolates with low-level elevated MICs to azithromycin (2.0 µg ml^−1^), carried a mosaic *mtr* operon and variants in PorB, and showed expansion with respect to data previously reported from 2018. The second largest clade predominantly carried the GyrA S91F variant, was largely ciprofloxacin resistant (MIC ≥1.0 µg ml^−1^), and showed significant expansion with respect to 2018. Overall, a low proportion of isolates had medium- to high-level elevated MIC to azithromycin ((≥4.0 µg ml^−1^), based on C2611T or A2059G 23S rRNA variants). One isolate carried the *penA* 60.001 allele resulting in elevated MICs to cefixime and ceftriaxone of 1.0 µg ml^−1^. This high-resolution snapshot of genetic profiles of 1710 GC sequences, through a comparison with 2018 data (1479 GC sequences) within the sentinel system, highlights change in proportions and expansion of select GC strains and the associated genetic mechanisms of resistance. The knowledge gained through molecular surveillance may support rapid identification of outbreaks of concern. Continued monitoring may inform public health responses to limit the development and spread of antibiotic-resistant gonorrhoea.

## Data Summary

All 1710 *Neisseria gonorrhoeae* sequence data files generated by a collaboration between the AR Lab Network laboratories and CDC for this study have been deposited in the National Center for Biotechnology Information Sequence Read Archive (NCBI SRA) (Project accession PRJNA317462, https://www.ncbi.nlm.nih.gov/bioproject/PRJNA317462/). BioSample IDs and SRA Accession IDs are provided in file 2019_GISP_alldata_supplemental.xlsx.

**Impact Statement**

*
Neisseria gonorrhoeae* (GC) is the bacterial pathogen that causes the sexually transmitted disease gonorrhoea. From 2009 to 2019 the reported cases of gonorrhoea in the USA increased 92 % to over 616 000 cases. This study characterized 1710 high-quality whole-genome sequences of GC isolates from 2019 collected as part of the USA Gonococcal Isolate Surveillance Project (GISP). This study provides a detailed report of strain diversity, phylogenetic relationships and resistance determinant profiles, and provides a side-by-side comparison with 2018 data to record the increased prevalence of strains of concern. The overall reported annual percent increase of isolates with elevated azithromycin (AZM) MICs within the USA from 2018–2019 was 0.5 % (from 4.6–5.1 %). This increase was due to an increase of isolates with MIC of 2.0 µg ml^−1^ (defined as nonsusceptible by CLSI breakpoints) and was explained by the continued expansion of one clade (represented by ST 9363, with mosaic *mtrRCDE* efflux pump operon). Analysis of the prominent *mtr* operon alleles within this clade, indicated recombination with other *Neisseria* species, and a different distribution of AZM susceptibility (MIC 1.0–2.0 µg ml^−1^) with the different alleles. The percentage of isolates with ciprofloxacin (CIP) resistance within the USA from 2018–2019 increased from 31.2–35.4 %, up 4.2 % (following the trend of previous years), even though CIP was not part of the recommended treatment regime for gonorrhoea since 2007. This increase from 2018–2019 was due to the expansion of one clade (represented by ST10314 and ST9902). Continued genomic surveillance as demonstrated with this 2019 GISP dataset will provide high-resolution details that impact the public health response to limit the development and spread of antibiotic-resistant gonorrhoea.

## Introduction

*Neisseria gonorrhoeae* (GC) is the bacterial pathogen that causes the sexually transmitted disease gonorrhoea. In 2019, 616 392 cases were reported to the USA Centers for Disease Control and Prevention (CDC) [1]. Symptomatic infections commonly occur as urethritis in men; pharyngeal, rectal or cervical infections are often asymptomatic. Additionally, GC can cause rare ocular infections and disseminated gonococcal infections (DGI) [2, 3]. Left untreated gonorrhoea can result in increased risk of contracting HIV, pelvic inflammatory disease, infertility, ectopic pregnancy, etc [4–6]. Strikingly, reported gonorrhoea cases have increased 92 % from 2009 to 2019 [7].

Given the ability of GC strains to quickly develop resistance to antibiotics used to treat infection, in 2019 the CDC continued to list antibiotic-resistant gonorrhoea as an urgent threat [8]. GC has developed resistance mechanisms to all antibiotics across multiple drug-classes that have been used for treatment in the USA since the first half of the twentieth century, including penicillin (PEN), tetracycline (TET), ciprofloxacin (CIP), azithromycin (AZM), cefixime (CFM) and ceftriaxone (CRO) [5]. In 2020 treatment guidelines for uncomplicated gonorrhoea changed from dual treatment with CRO and AZM to monotherapy with a higher dose of CRO alone partially due to the increased prevalence of isolates with reduced susceptibility to AZM [9]. A variety of resistance mechanisms, often resulting from chromosomal mutations (single-nucleotide polymorphisms or recombination events) and plasmid-encoded determinants that can confer antibiotic resistance have been utilized by the gonococcus, resulting in decreased import of drugs into the cell (e.g. *porB1b* mutations), increased export through efflux pumps (e.g. *mtrCDE* multi-drug efflux pump), inhibition of interaction with or decreased affinity for the drug target (e.g., 23S rRNA, *penA* or *gyrA/parC*), and enzymes that inactivate the drug (e.g. β-lactamase) [5, 10]. As understanding of the role of genetic exchange with *Neisseria meninigitidis* and commensal *Neisseria* species increases, it becomes apparent that a reservoir of resistance mechanisms may be available to GC necessitating that we continue to find new ways to fight drug resistance [11]. GC will readily undergo homologous recombination to incorporate DNA from other *Neisseria* species into loci such as *penA* and the *mtrRCDE* operon, resulting in mosaic alleles that often reduce the bacteria’s susceptibility to CRO and AZM, respectively [12–15].

To monitor GC antibiotic resistance trends in the USA, the Gonococcal Isolate Surveillance Project (GISP) was established in 1986 at the CDC. GISP collects the first 25 GC isolates per month from men with symptomatic urethral gonococcal infections visiting each participating sentinel, clinic site [16]. In 2017, supported by the Combating Antibiotic Resistant Bacteria (CARB) initiative, in addition to performing antibiotic susceptibility testing on all GISP isolates, the GC Antibiotic Resistance Laboratory Network (AR Lab Network) regional laboratories began performing whole-genome sequencing (WGS) on the first five GISP isolates per month, with 2018 being the first full year the data were collected [17–19]. This allowed for a higher resolution of GC strain presence and understanding and monitoring of molecular mechanisms of antibiotic resistance. Comparisons over time and between jurisdictions may provide insight into emerging strains, evolution of resistance determinants, and targeted public health actions. Here we present the data from WGS of the first five GISP isolates collected per month in 2019. We observed the presence of various sequence types in this dataset, looked at the phylogenetic relationships of the isolates, assessed the genetic antimicrobial resistance markers associated with elevated MICs to antibiotics, and made comparisons to the trends observed in 2018.

## Methods

### Isolate collection and AST, 2019

As part of GISP, in 2019, clinical specimens were collected from the first 25 symptomatic, male gonococcal urethritis cases attending clinics across 32 participating, sentinel sites. GC was isolated from clinical specimens, and antimicrobial susceptibility testing (AST) using the agar dilution method was performed at AR Lab Network regional laboratories following GISP protocols (https://www.cdc.gov/std/gisp/gisp-egisp-protocol-august-2021.pdf) [17]. MICs were determined for CIP, TET, PEN, AZM, CFM, CRO and gentamicin (GEN). MICs used as cutoffs for our analyses (as set by GISP) are shown in Table 1 in comparison to available CLSI breakpoints [7, 20]. An MIC cutoff one dilution lower than or equal to the CLSI susceptibility breakpoint (i.e. MIC 0.25 µg ml^−1^) for CRO or CFM, respectively, was used to detect developing resistance and for consistency with other GISP analyses and reports. Furthermore, due to the lack of CLSI resistance breakpoints and for consistency with GISP [7], for this study MICs for CRO, CFM and AZM that were at or above their respective designated cutoffs of concern will be referred to as ‘elevated MIC’ (em) throughout the manuscript. There is currently no CLSI susceptibility or resistance breakpoint set for GEN; there were no isolates in the dataset with GEN MIC ≥32 µg ml^−1^.

**Table 1.** Antibiotic MIC cutoff values for analysis and CLSI breakpoints

Antibiotic	CLSI susceptibility breakpoint∗	CLSI resistance Breakpoint∗, †	MIC value‡ of study
Ceftriaxone §	≤ 0.25 µg ml^−1^		≥ 0.125 µg ml^−1^
Cefixime §	≤ 0.25 µg ml^−1^		≥ 0.25 µg ml^−1^
Azithromycin	≤ 1.0 µg ml^−1^		≥ 2.0 µg ml^−1^
Ciprofloxacin	≤ 0.06 µg ml^−1^	≥ 1.0 µg ml^−1^	≥ 1.0 µg ml^−1^
Penicillin	≤ 0.06 µg ml^−1^	≥ 2.0 µg ml^−1^	≥ 2.0 µg ml^−1^
Tetracycline	≤ 0.25 µg ml^−1^	≥ 2.0 µg ml^−1^	≥ 2.0 µg ml^−1^

*Susceptibility and resistance breakpoints set by the Clinical and Laboratory Standards Institute (CLSI) 2022 [20].

†No CLSI resistance breakpoints have been determined for ceftriaxone, cefixime or azithromycin.

‡MIC values of concern used for analysis in this study and by GISP in 2019 [7].

§An MIC cutoff one dilution lower than or equal to the CLSI susceptibility breakpoint for CRO or CFM, respectively, was used to aid attempts to detect developing resistance [7, 20].

### Whole-genome sequencing of select isolates

The first five GISP isolates per jurisdiction per month were selected for WGS following CDC guidance (as described in GISP protocols: https://www.cdc.gov/std/gisp/gisp-egisp-protocol-august-2021.pdf), which was performed by AR Lab Network regional laboratories based on PulseNet protocols (https://www.cdc.gov/pulsenet/pathogens/wgs.html) for DNA extraction, DNA library preparation and Illumina sequencing on MiSeq with either V2 or V3 chemistry (Illumina, San Diego, CA). WGS raw reads were transferred to CDC for assembly, quality control and analytical processing. Sequence data are available in the National Center for Biotechnology Information Sequence Read Archive (NCBI SRA) (Project accession PRJNA317462).

The whole-genome sequences of isolates from GISP 2018, used in comparison in this study, were as published in [19] and are available in NCBI SRA (Project accession PRJNA317462). References are also made to the whole-genome sequences of isolates from GISP 2017, which are published in [18] and are available in NCBI SRA (Project accession PRJNA317462).

### Bioinformatic analyses

WGS bioinformatic processing and analyses followed previously published protocols [18, 19, 21].

Briefly, pre-processing steps included a contaminant assessment (Kraken v0.10.5) [22] where the percent *Neisseria gonorrhoeae* versus all bacterial reads and percent *N. gonorrhoeae* or *N. meningitidis* were measured. Trimming was performed using (Cutadapt v1.8.3, 1.16) [23] to remove adaptor sequences and bases using a minimum quality cutoff of Q30, and read quality assessment and percent duplication were determined using FastQC 0.11.5 (http://www.bioinformatics.babraham.ac.uk/projects/fastqc/). Genomes were assembled with SPAdes Assembler v3.9.0 [24] using default parameters. Quality assessment statistics were monitored and reported throughout the analysis. quast v4.3 [25] was used for assembly statistics including the number of contigs, N50, percent genome assembled, percent G-C; in addition, final genome assembly size and depth of coverage was measured through multiple means. Required assembly statistics for inclusion in the study are provided in Table S1B, available with the online version of this article.

*In silico* sequence typing was performed using Multilocus Sequence Typing (stringMLST v0.3.6) [26], *N. gonorrhoeae* multi-antigen sequence typing (NG-MAST) (ngmaster v0.4) [27], and *N. gonorrhoeae* sequence typing for antimicrobial resistance (pyngSTar, https://github.com/leosanbu/pyngSTar) [28]. For novel alleles and sequence types, assignments were made after submission of the genomes to the respective databases for MLST and NG-MAST [https://pubmlst.org/bigsdb?db=pubmlst_neisseria_seqdef, MLST 31 January 2022 and NG-MAST 2 June 2022) and NG-STAR (https://ngstar.canada.ca/, 31 January 2022)] [28]. Sequence typing is a technique that characterizes the DNA sequence variation in a set of defined gene loci by their unique allelic profiles. MLST is based on seven house-keeping genes (constitutive genes required for cellular function); NG-MAST is based on two ‘highly variable’ and polymorphic outer membrane protein genes (porB and tbpB); and NG-STAR is based on seven genes associated with resistance to beta-lactam antimicrobials, macrolides and/or fluoroquinolones.

Whole-genome alignment was performed using bwa/0.7.12 [29], samtools/1.3.1 [30] and/or freebayes/1.0.2 (https://github.com/freebayes/freebayes) for mapping of gene loci and genomic variants. Whole-genome alignment for phylogenetic reconstruction was generated using snippy v4.3.8 (https://github.com/tseemann/snippy) with FA19 (GenBank accession number CP012026.1) as the reference and the recombination events were detected and filtered using Gubbins v2.3.2 [31]. The resulting alignment, containing only the polymorphisms present in the nonrecombinant regions, was used as input for RAxML v8.2.9 under the GTR+GAMMA model of nucleotide substitution with a majority-rule consensus (MRE) convergence criterion, to reconstruct an ascertainment bias corrected (Stamatakis method) maximum-likelihood (ML) phylogeny [32]. Whole-genome phylogenetic analysis was performed for the 2019 dataset (*n*=1710), and for a combined 2018–2019 dataset (*n*=3189). Each clade, within the phylogenetic tree, is defined as having a common ancestral origin and represents a monophyletic group of lineal descendants. Clade identification was calculated using fastbaps v1.0.4 with optimize prior and optimized.symmetric options [33], and visualized via Interactive Tree of Life (iTOL) [34] or using the R package ggtree [35]. The reference for assembly, mapping and phylogenetics was GC strain FA19 (GeneBank accession CP012026.1). A custom CDC programme, AMR-Profiler and Typing Tool 2.8.3/2.9.2, identified the sequence per specimen at 75 genomic loci determined to be of interest based on known associations with antimicrobial resistance (AMR) (including chromosomal and plasmid genes; variants discussed in the manuscript are listed in Table S2) from raw sequence reads and assemblies [21].

### *mtr* operon allele analysis

A local blast database was generated from the 1710 contigs of this 2019 GISP WGS dataset. A query sequence of the *mtrRCDE* operon from strain FA19 (GenBank accession CP012026.1, nucleotide positions 1104741–1111533) was searched against the local database via blastn (ncbi-blast-2.9.0+; https://blast.ncbi.nlm.nih.gov/Blast.cgi). The blast results from the sequences in fastbaps clade 20 (*n*=372) were aligned with the FA19 *mtr* operon sequence using mafft (version 7.471) [36]. Subclades were determined from this alignment using fastbaps v1.0.4 [33]. mafft was then used to separately align the sequences in each major subclade (1, 2, 5 and 6), and CLC Genomics Workbench 20.0.2 (https://digitalinsights.qiagen.com/) was used to visualize the alignments and perform pairwise comparisons. A representative sequence (the sequence most prevalent in each clade) from each individual subclade alignment (of the sequences in the subclades 1, 2, 5 and 6) was used in an alignment together along with the *mtr* operon sequence from CP012026.1 (genome positions 1104741–1111533) and visualized in the NCBI Multiple Sequence Alignment Viewer 1.21.0 (https://www.ncbi.nlm.nih.gov/tools/msaviewer/). Additional genomic analyses (including identification of *mtrC* and *mtrD* premature stop codons), alignments and generation of an *mtr* operon neighbour-joining tree with the Kimura 80 substitution model were performed in CLC Genomics Workbench 20.0.2. The four subclade representative sequences, as well as the positions corresponding to the *mtrD* gene, were used in blastn queries against the NCBI database to determine what species were most similar to the queries.

Whole-genome alignment for clade 20 isolates (*n*=372) was also generated using snippy with FA19 (CP012026.1) as the reference and the putative recombination events were detected using Gubbins v2.3.2 with minimum SNPs to identify a recombination block set to ten SNPs. The inferred recombination events at the *mtr* operon region (with 1000 basepairs upstream and downstream) integrated with the *mtr* operon neighbour-joining tree described above was visualized using the R package RCandy [37].

### Statistical analyses

Statistical analyses were conducted using R (v. 4.0.4) and RStudio Server (v. 1.3.1073) software. Hypothesis testing for percentage of isolate MLST between 2019 and 2018 were analysed using Fisher’s exact test with mid-*P* values from the exact 2×2 R package (https://cran.r-project.org/web/packages/exact2x2/index.html). Analysis of clades and subclades by patients’ sex of sex partner was tested using Fisher’s exact test with mid-*P* values. Sex of sex partners was categorized as follows: men who have sex with women only (MSW), men who have sex with men only (MSM), men who have sex with men and women (MSMW) and unknown. The proportion of isolates per antimicrobial resistance MIC category between 2019 and 2018 were analysed using Pearson’s Chi-squared test, base R, without continuity correction. All hypothesis testing used a significance level of α=0.05.

## Results

In 2019, there were 5480 isolates collected from 32 sentinel sites participating in GISP (up to 25 isolates/month/jurisdiction) [7]. Of the first five of those isolates per month collected from each jurisdiction, a total of 1710(96 %, 1710/1777) were successfully sequenced. The remainder were not available, non-viable at the AR Lab Network, or did not provide a high-quality sequence after multiple attempts. Of the 32 sentinel sites, 26 sites had >80 % of their first five isolates/month sequenced for the year. The remaining 6 sites had only 55–75 % of the potential first five isolates sequenced. (See File S1: 2019_GISP_alldata_supplemental.xlsx).

### Demographics

**Fig. 1.**
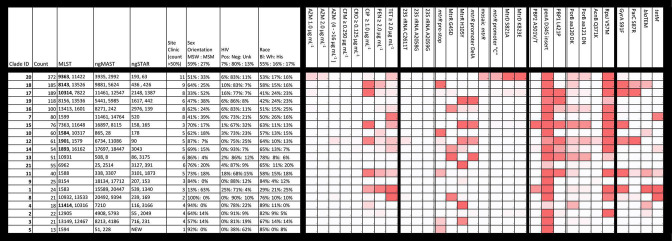
Phylogenetic clades with associated genotypic, demographic and phenotypic characteristics for GISP 2019 first five isolates (*n*=1710). Monophyletic clades were calculated by fastbaps and are defined by having a common ancestral stem. The number of isolates per fastbaps clade along with the most prevalent sequence types. The heat map highlights the proportion of isolates with antimicrobial resistance (AMR) variants (high proportion of variant [>75 %, red] [50–75 % variant, pink], low proportion of variant [25–50 %, light pink], no variant [white]). AMR variants: *mtrR* promoter=adenine nucleotide deletion (Del A); *mtrR* promoter ‘C’ = adenine to cytosine variant (A>C); PBP2 (penA) A501V/T=Val or Thr variant in position 501; PorB aa120DK=Asp or Lys variant in position 120; PorB aa121DN=Asp or Asn variant in position 121; *bla*_TEM_ or tetM=the presence of the plasmid-based gene.

The isolates were collected from patients representing a broad range of ages, races/ethnicities, and sex of sex partner. The age, race, ethnicity, and sex of sex partners of patients (as directly ascertained by clinicians during patient interview) from whom GC isolates were collected for AST in GISP were similar to the characteristics of patients of the subset of isolates that were sequenced (Table 2). The predominant age group was 25–29 year olds which accounted for 24 % (404/1710) of patients. Based on patient reported sex of recent sex partners, 59 %(1001/1710) of patients were categorized as MSW, 27 % (469/1710) as MSM and 6 % (108/1710) were MSMW. The predominant race/ethnicities were 55 % (948/1710) Black, 17 % (289/1710) Hispanic and 16 % (278/1710) White (Table 2, Fig. 1).

**Table 2.** Patient demographics associated with GC isolates, GISP 2019 Counts for all GISP isolates in 2019 [7] versus GISP isolates that were sequenced.

	AST (*n*=5480)		WGS (*n*=1710)	
Age	Count	Percent	Count	Percent
≤19	326	6 %	98	6 %
20–24	1079	20 %	318	19 %
25–29	1365	25 %	404	24 %
30–34	952	17 %	313	18 %
35–39	585	11 %	196	11 %
40–44	378	7 %	126	7 %
45–49	273	5 %	82	5 %
50–54	210	4 %	70	4 %
55–59	169	3 %	57	3 %
60–64	77	1 %	25	1 %
≥65	48	1 %	20	1 %
Unknown	18	0.3 %	1	0.1 %
**Sex of sex partners**				
MSW	3436	63 %	1002	59 %
MSM	1443	26 %	469	27 %
MSMW	324	6 %	108	6 %
Unknown	244	4 %	125	7 %
**Race/ethnicity**				
Black	3132	57 %	948	55 %
White	927	17 %	278	16 %
Hispanic	894	16 %	289	17 %
Asian	118	2 %	49	3 %
Multi-racial	90	2 %	33	2 %
Native Hawaiian	22	0.4 %	14	0.8 %
American Indian	41	0.7 %	22	1 %
Other	102	2 %	32	2 %
Unknown	154	3 %	45	3 %

AST, antimicrobial susceptibility testing; MSM, men who have sex with men only; MSMW, men who have sex with men and women; MSW, men who have sex with women only; WGS, whole-genome sequencing.

### Molecular strains

**Fig. 2.**
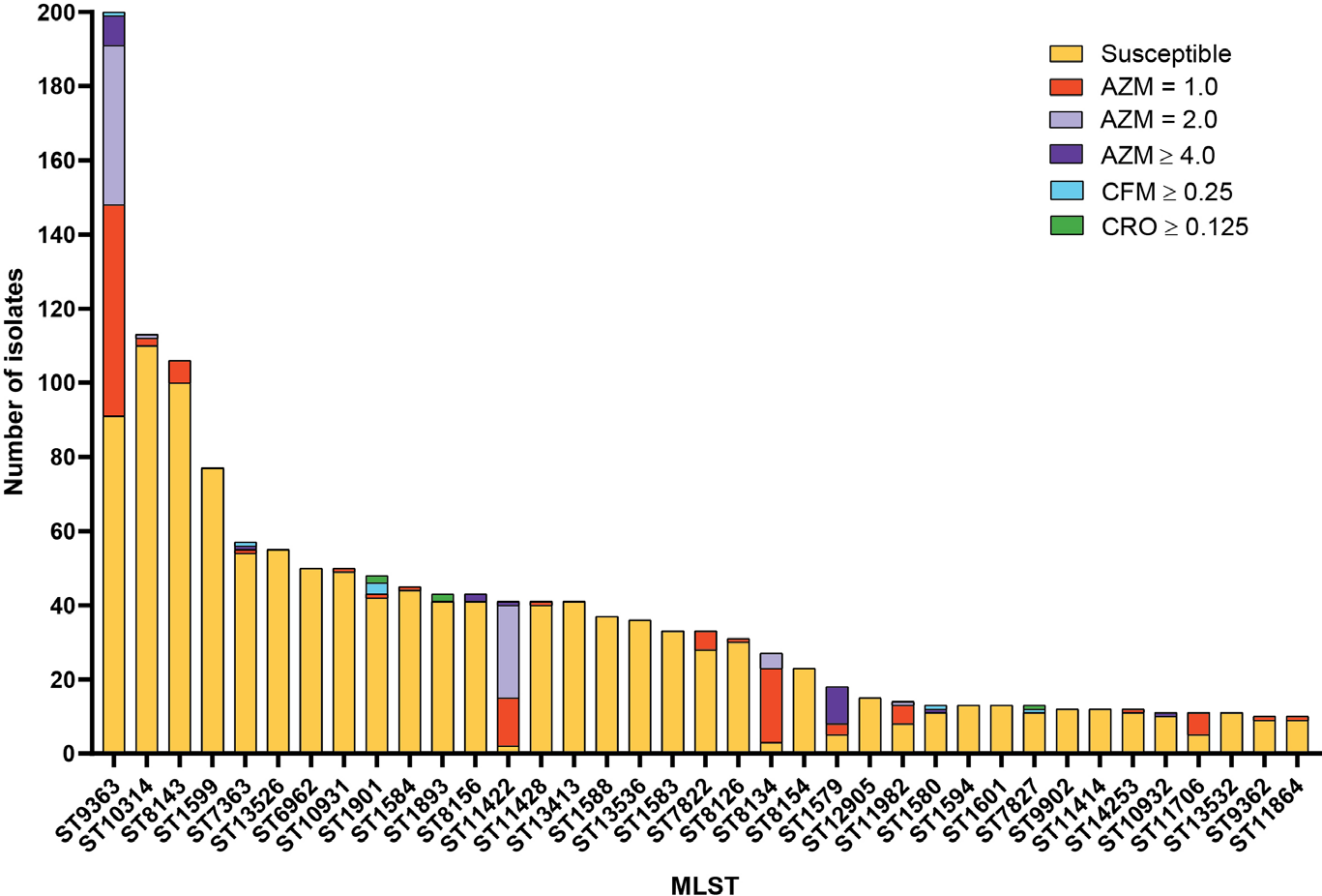
Multilocus sequence type (MLST) distribution of the 1710 GISP first five isolates from 2019 in the USA. The 37 most prevalent STs (that include >10 isolates) are shown. Proportions of isolates are coloured by MIC to AZM (MIC 1.0 µg ml^−1^ red, MIC 2.0 µg ml^−1^ light purple, MIC ≥4.0 µg ml^−1^ dark purple), CFM (MIC ≥0.25 µg ml^−1^ cyan) and CRO (MIC ≥0.125 µg ml^−1^ green). MIC=minimum inhibitory concentration; AZM=azithromycin; CFM=cefixime; CRO=ceftriaxone.

Multi-locus sequence typing (MLST) [38], *Neisseria gonorrhoeae* multiantigen sequence typing (NG-MAST) [39], and *N. gonorrhoeae* sequence typing for antibiotic resistance (NG-STAR) [28] were used to identify the sequence types. The 1710 isolates were represented by 164 different MLST types. Thirty-seven sequence types (STs) included ≥10 isolates per ST and accounted for 82.9 % (1418/1710) of all isolates (Fig. 2). Twenty-five STs were represented by only two isolates each, and 69 STs (42 %, 69/164) included only one isolate (see File S2: 2019_GISP_alldata_06062022.xlsx). The most predominant MLST STs were ST9363 (200/1710, 11.7 %), ST10314 (113/1710, 6.6 %), ST8143 (106/1710, 6.2 %), ST1599 (77/1710, 4.5 %), ST7363 (57/1710, 3.3 %) and ST13526 (55/1710, 3.2 %).

In this 2019 first five dataset, several STs represented a higher percentage than measured in the previous 2018 GISP first five dataset [19] (Tables 3 and S3). ST10314 increased from representing 3.3 % (49/1479) of the isolates (eighth most common) in 2018 to 6.6 % (113/1710) (second) in 2019 (*P* value 1.89 e^−05^) (Table 3). ST9363 showed a non-significant increase from 10.7 % in 2018 to 11.7 % in 2019 (remaining as the most predominant), ST8126 increased from 0.5 to 1.8 % (*P* value 3.6 e^−04^); ST8134 increased from 0.5 % (7/1479) to 1.6 % (27/1710) (*P* value 2.0 e^−03^). ST1584, one of the top represented STs in 2018, decreased from 4.0 % (third) in 2018 to 2.6 % (tenth) in 2019 (*P* value 3.2 e^−02^).

**Table 3.** Comparison of the percentages of MLSTs among the GISP first five isolates in 2019 vs. 2018*

MLST	Count in 2018†	Percent in 2018†	Count in 2019	Percent in 2019	*P* value
**Increased in 2019**					
10 314	49	3.31 %	113	6.61 %	1.8905 e^−05^
8126	7	0.47 %	31	1.81 %	3.5970 e^−04^
8134	7	0.47 %	27	1.58 %	2.0304 e^−03^
11 982	3	0.20 %	14	0.82 %	1.665 e^−02^
9902	3	0.20 %	12	0.70 %	4.134 e^−02^
**Decreased in 2019**					
1584	59	3.99 %	45	2.63 %	3.239 e^−02^
1601	26	1.76 %	13	0.76 %	1.135 e^−02^
11 414	22	1.49%	12	0.70 %	3.345 e^−02^
8149	12	0.81 %	5	0.29 %	4.999 e^−02^
12 462	14	0.95 %	3	0.18 %	3.02 e^−03^
11 516	8	0.54 %	2	0.12 %	3.899 e^−02^
11 181	10	0.68 %	1	0.06 %	3.10 e^−03^
7371	7	0.47 %	1	0.06 %	2.393 e^−02^
1585	7	0.47 %	1	0.06 %	2.393 e^−02^
13 489	6	0.41 %	1	0.06 %	4.638 e^−02^

*Only the comparisons with statistically significant differences are shown. All comparisons are listed in Table S3.

†The whole-genome sequences of isolates from GISP 2018, used in comparison in this study, were as published in [19].

GISP, Gonococcal Isolate Surveillance Project; MLST, multilocus sequence typing.

The 1710 isolates were distributed among 515 NG-MAST types (with additional 321 novel *porB* and 94 novel *tbpB* sequences) and 408 NG-STAR types (50 novel types). The most common NG-MAST types in 2019 were ST11461 (*n*=63), ST3935 (*n*=54), ST865 (*n*=27), ST9918 (*n*=24) and ST508 (*n*=21); the most common NG-STAR types were ST193 (*n*=77), ST436 (*n*=76), ST520 (*n*=73), ST63 (*n*=64), ST2148 (*n*=56), ST178 (*n*=48) (Figs 1, S1A, B).

### Phylogenetics

**Fig. 3.**
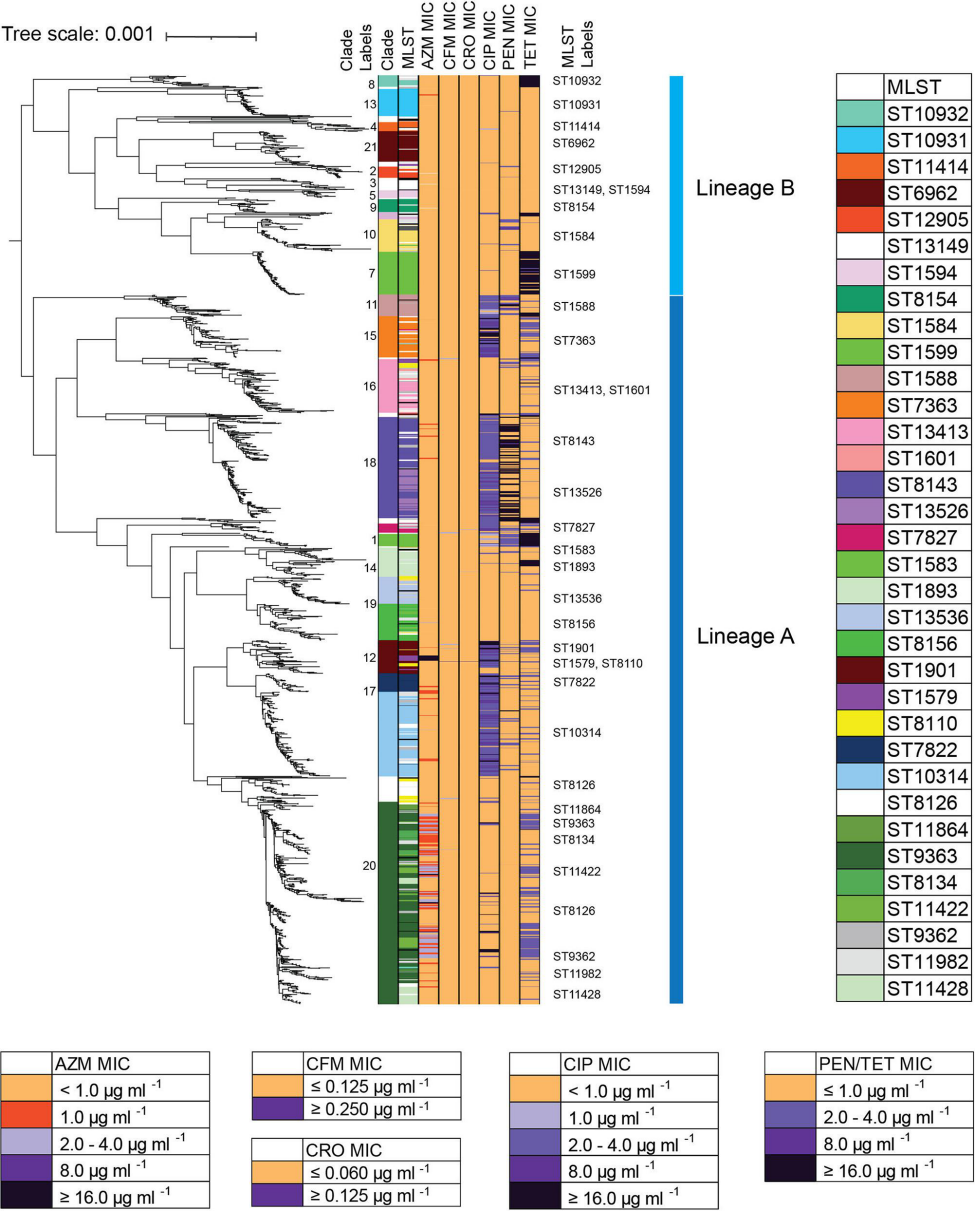
Maximum-likelihood core-genome SNP phylogenetic reconstruction of 1710 GISP first five isolates from the USA in 2019. Maximum-likelihood core-genome phylogenetic analyses defined the 1710 isolates into two lineages, A and B, and into 21 clades (‘Clade Label’ left column) and color-defined in the left-most column. Isolates that were not grouped into a predominant phylogenetic clade are uncolored (white). MLST STs are shown (with a colour key to the right, listed approximately in order of appearance) and labelled (‘MLST Label’ in centre column). MLSTs with low representation are uncolored (white). Isolate susceptibility profiles are shown for AZM, CFM, CRO, CIP, PEN and TET and coloured according to MIC [susceptible (gold), elevated MIC (shades of purple)].

A maximum-likelihood core-genome SNP phylogenetic analysis categorized the isolates into 21 major clades (defined by having a common ancestral stem) (Fig. 3) and two major lineages as described by Sanchez-Buso [40].

Lineage A accounted for 76.4 % (1307/1710) of all of the isolates, and 86.6 % (817/943) of the isolates that had resistance or an elevated MIC (as defined by the cutoffs in Table 1) to one or more antibiotics. Only 37.4 % (489/1307) of Lineage A isolates are susceptible/nonresistant to all antibiotics (as defined as an MIC below the cut-offs in Table 1). Overall, 70.1 % (343/489) of the susceptible/nonresistant isolates of Lineage A group in clades 20.1 [ST9363, ST11422 (*n*=152)], 19 [ST8156, ST13536 (*n*=112)] and 16 [ST1601 (*n*=79)].

Lineage B accounted for 23.6 % (403/1710) of all of the isolates, and 13.3 % (125/943) of the isolates that had resistance or an elevated MIC to one or more antibiotics. 69.0 % (278/403) of Lineage B isolates were susceptible/nonresistant to all antibiotics. 25.6 % (103/403) of lineage B was resistant to TET, and less than 5 % were resistant to PEN (4.7 % (19/403)) or CIP (2.2 % (9/403)). Only one isolate had an elevated MIC for AZM (≥ 2.0 µg ml^−1^).

**Fig. 4.**
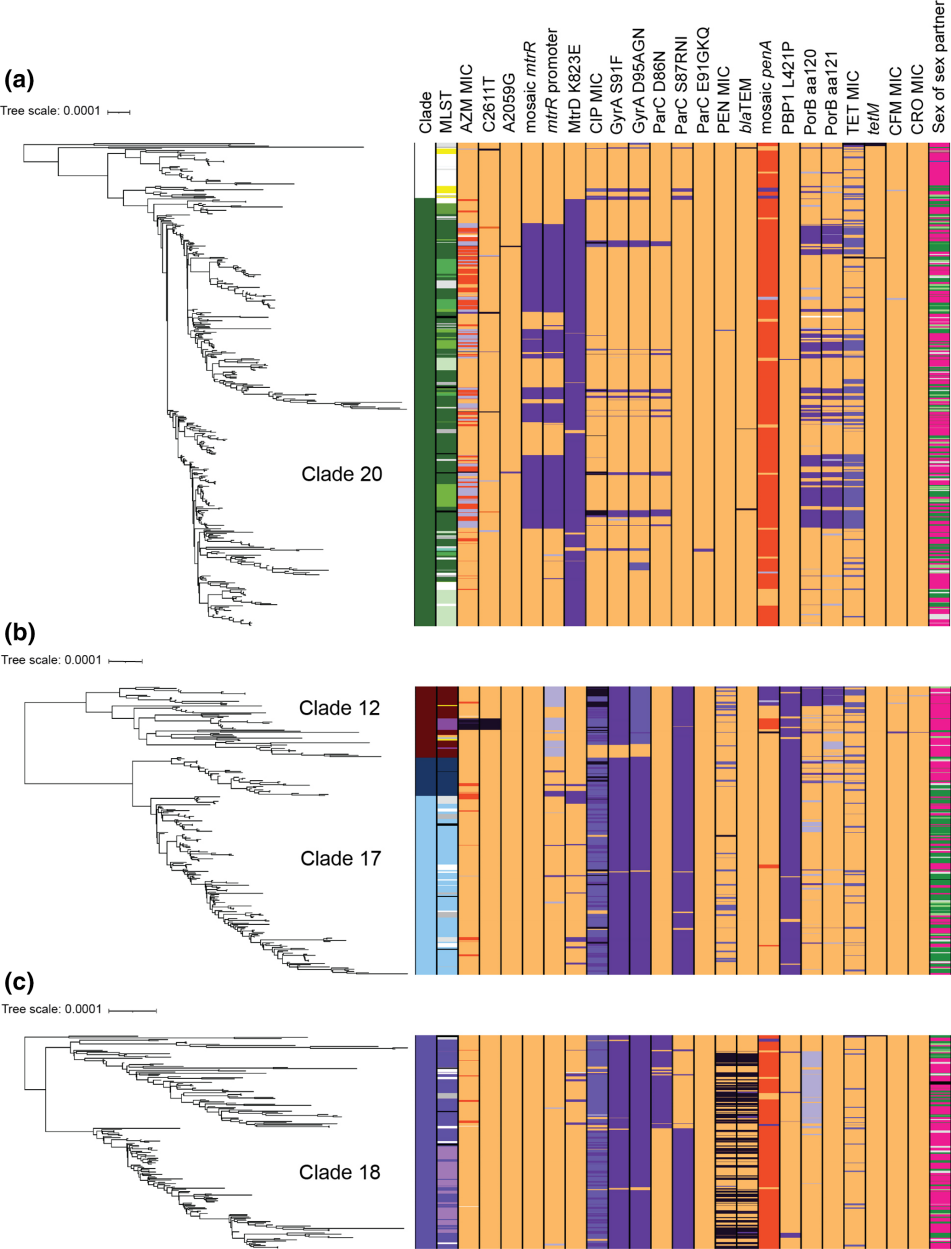

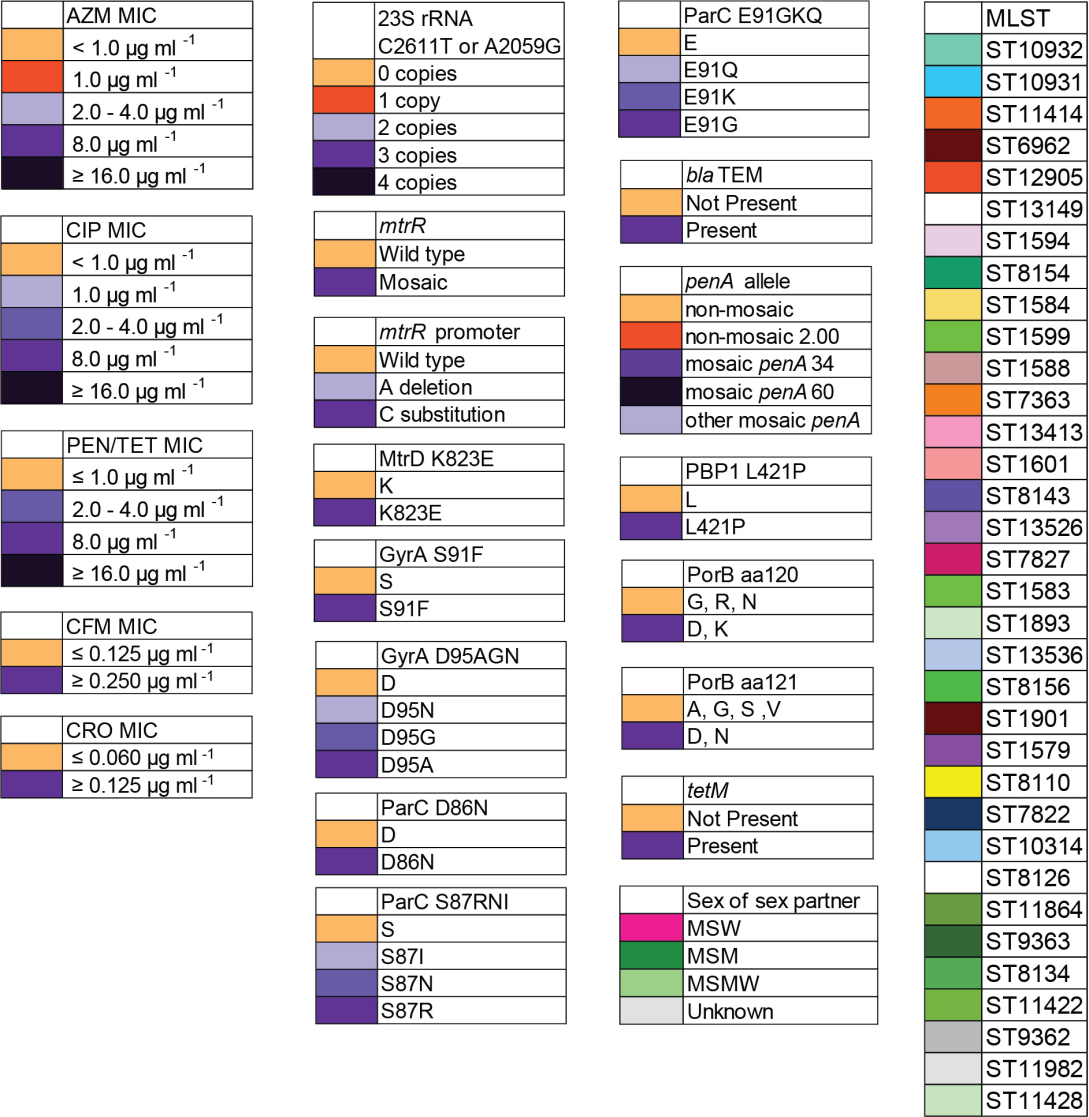
Clade-specific maximum-likelihood core-genome SNP phylogenetic reconstruction. Antibiotic susceptibility profiles and genetic variant profiles are specified for (a) clade 20(372/1710 isolates), (b) clade 17(189/1710 isolates) and clade 12(61/1710) and (c) clade 18(185/1710 isolates). Isolate susceptibility profiles are shown for AZM, CIP, PEN, TET and CFM/CRO, and coloured according to MIC; with gold for susceptible and shades of purple for elevated MIC (AZM, CFM, CRO) or resistant (CIP, PEN, and TET). The variants are represented as wild-type (gold) or mutant (orange, or light to dark purple). Sex of sex partner is represented as pink for men who have sex with women only, dark green for men who have sex with men only, light green for men who have sex with men and women, and grey for unknown.

Of the 21 major clades (Figs 1 and 3), the largest was clade 20 (Fig. 4), accounting for 21.7 %(372/1710) of the isolates, and carrying 84.1 % (85/101) of the isolates with AZM MIC ≥2.0 µg ml^−1^ (AZM^em^), though only 22.8 %(85/372) of the clade has AZM^em^. Altogether, 37.6 % (140/372) of isolates in clade 20 were resistant to TET (MIC ≥2.0 µg ml^−1^, TET^R^), and a small percent (8.3%, 31/372) carried CIP resistance (MIC ≥1.0 µg ml^−1^, CIP^R^). As seen in 2018 (then designated as clade 16), this clade was predominately MLST ST9363 (53.8%, 200/372), ST11422 (11.0%, 41/372), ST11428 (11.0%, 41/372) and ST8134 (7.2 %, 27/372) [19]. From 2018–2019 the overall percent of ST9363 increased, albeit insignificantly (Table S3); at the same time, statistically significant increases in percentages were observed for ST8134 and ST11982 (Table 3). The relationship between 2018 and 2019 lineages and the expansion of this clade from 19 % (278/1479) in 2018 to 22 % (372/1710) in 2019 can be seen in the phylogenetic tree generated with the merged dataset (*n*=3190) (Figs S2A and S2B). Clade 20 represented a patient population of 51 % (188/372) MSW and 33 % (123/372) MSM; 53 % (196/372) Black, 17 % (62/372) White and 16 % (61/372) Hispanic (Fig. 1).

The second largest clade, 17, included 11.0 % (189/1710) of isolates which were found in ST10314 (59.8%, 113/189), ST7822 (17.5 %, 33/189), and ST9902 and ST14253 (each at 6.3 %, 12/189) (Figs 1 and 4b). From 2018–2019 ST10314 showed the highest increase from 3.3–6.6 % (49/1479 to 113/1710, *P* value 1.9 e^−05^), and ST9902 increased from 0.20–0.70 % (3/1479 to 12/1710, *P* value 4.1 e^−02^) (Table 3). The expansion of this clade from 6 % (96/1479) in 2018 to 11 % (189/1710) in 2019 can be seen in the phylogenetic tree (Fig. S2C). This clade is associated with CIP^R^ (96.2 %, 182/189), and a small percent with chromosomally-based PEN^R^ and TET^R^ [12.7 % (24/189) and 13.7 % (26/189), respectively]. Clade 17 represented a patient population significantly over-represented by MSM (52 %, 99/189, *P* value 1.1 e^−14^) with 33 % (63/189) MSW and 41 % (78/189) Black, 24 % (45/189) White, and 23 % (43/189) Hispanic (Fig. 1).

Clade 18 included 10.8 % (185/1710) of the isolates, predominately in ST8143 (57.3 %, 106/185) and ST13526 (29.7 %, 55/185). A majority of the isolates were CIP^R^ (94.0 %, 174/185) (NG-STAR ST436, ST426), and 48.6 %(87/179) carried PEN^R^ (49.1 %, 91/185) associated with presence of the *bla*_TEM_ gene (Fig. 4c). Clade 18 showed no increase in percentage of sequenced isolates from 2018 to 2019. The lineage of this clade represented by 11 % in both 2018 (11 %, 159/1479) and 2019 (11 %, 185/1710) can be seen in the phylogenetic tree (Fig. S2D). Clade 18 represented a patient population of 64 % (119/185) MSW and 25 % (46/185) MSM (similar to the overall population) [7]. Patients were 58 % (108/185) Black, 15 % (27/185) White, 16 % (29/185) Hispanic (Fig. 1).

Clade 12, the ninth largest clade, accounted for 3.6 %(61/1710) of isolates and included MLST ST1901, ST1579 and ST8110 (Fig. 4b). Historically in the USA GISP genomic reports (years 2014–2019), ST1901 (and phylogenetically related sequence types ST9365 and ST8110) included isolates with elevated MIC to CFM (as defined by the cutoffs in Table 1, MIC≥0.25 µg ml^−1^, CFM^em^) and to CRO (as defined by the cutoffs in Table 1, MIC≥0.125 µg ml^−1^, CRO^em^) (Table S9B) [18, 19, 21]. In 2019, 3 of 8 isolates with CFM^em^ were in ST1901, including the one isolate with CFM MIC 1.0 µg ml^−1^. Two of the five isolates with CRO^em^ were also in this ST. ST1901 also included isolates with resistance to CIP (82.0 %, 50/61), PEN (19.7 %, 12/61), and TET (37.7 %, 23/61). Clade 12 represented a patient population of 87 % (53/61) MSW and 7 % (4/61) MSM (which represents statistically higher percent MSW than the overall dataset, p-value 1.3 e^−06^); 64 % (39/61) Black, 10 % (6/61) White, and 13 % (8/61) Hispanic (Fig. 1).

### Antimicrobial resistance determinants and susceptibilities

Overall, 44.9 % (767/1710) of isolates were susceptible/nonresistant to all antibiotics studied (CIP, TET, PEN, AZM, CFM and CRO) as defined by either [1] having MIC below the CLSI resistance breakpoint (CIP, TET, PEN) or [2] having a MIC considered susceptible based on the CLSI susceptibility breakpoint (AZM) or [3] having an MIC cutoff one dilution lower than (CRO <0.125 µg ml^−1^) or equal to (CFM<0.250 µg ml^−1^) the CLSI susceptibility breakpoint as defined in other GISP analyses (Table 4). Overall, 55.1 % (943/1710) of isolates had elevated MICs above these cutoffs to one or more antibiotics. In total, 22.6 % (387/1710) had elevated MICs to two or more antibiotics. There were no isolates with elevated MICs to all drugs tested, and no isolates with both AZM^em^ and CRO^em^ (the two drugs of treatment in 2019). These counts were representative of the patterns of resistance or patterns of elevated MIC reported in GISP 2019 (Table 4) [7].

**Table 4.** Combinations of MIC profiles among GISP isolates in 2019 Counts for all GISP in 2019 (AST) [7] versus GISP isolates that were sequenced (WGS).

	AST (*n*=5480)		WGS (*n*=1710)		
**Antibiotic(s)**	**Count**	**Percent**	**Count**	**Percent**	
Susceptible/nonresistant^*^	2440	44.5 %	767	44.9 %	
**CIP**	1003	18.3 %	299	17.5 %	
**TET**	743	13.6 %	211	12.3 %	
**PEN/CIP**	330	6.0 %	115	6.7 %	
**TET/CIP**	324	5.9 %	108	6.3 %	
**PEN/TET/CIP**	196	3.6 %	52	3.0 %	
**TET/AZM**	146	2.7 %	50	2.9 %	
**PEN**	91	1.7 %	24	1.4 %	
**AZM**	60	1.1 %	19	1.1 %	
**PEN/TET**	50	0.9 %	23	1.3 %	
**TET/CIP/AZM**	44	0.8 %	18	1.1 %	
**CIP/AZM**	25	0.5 %	10	0.6 %	
**PEN/TET/ESC/CIP**	12	0.2 %	5	0.3 %	
**ESC**	8	0.1 %	3	0.2 %	
**TET/ESC/CIP**	3	0.1 %	2	0.1 %	
**PEN/TET/CIP/AZM**	3	0.1 %	2	0.1 %	
**PEN/TET/AZM**	1	0.0%	1	0.1 %	
**ESC/CIP**	1	0.0 %	1	0.1 %	

*Susceptible/nonresistant: CIP, TET and PEN MICs below the CLSI resistance breakpoints (CIP <1.0 µg ml^−1^, TET <2.0 µg ml^−1^, PEN <2.0 µg ml^−1^); AZM (≤1.0 µg ml^−1^) based on the CLSI susceptibility breakpoint for AZM; CRO and CFM MICs (CRO<0.125 µg ml^−1^, CFM<0.25 µg ml^−1^) based on an MIC cutoff one dilution lower than or equal to the CLSI susceptibility breakpoint for CRO and CFM, respectively [7, 20].

AST, antimicrobial susceptibility testing; ESC, extended-spectrum cephalosporins (Ceftriaxone and/or cefixime); WGS, whole-genome sequencing.

### Azithromycin

Out of 1710 isolates in the dataset, 101 (5.9 %) had an elevated AZM MIC of ≥2.0 µg ml^−1^ [as defined as an MIC above the CLSI susceptibility cutoff, AZM^em^ (Table 1)]. Combinations of resistance determinants relevant to the range of AZM MICs of these isolates are found in Table 5. In total, 75.2 % (76/101) of these isolates had low-level AZM^em^ (2.0 µg ml^−1^), and 24.7 %(25/101) had medium to high-level AZM^em^ (≥4.0 µg ml^−1^). From 2018–2019, the percent of isolates with AZM MIC 2.0 µg ml^−1^ increased by 1.4 % (*P* value 3.9 e^−02^); more significantly the percent of isolates with AZM MIC 1.0 µg ml^−1^ increased by 3.2 % (*P* value 1.0 e^−04^); the percent of isolates with medium to high level AZM MIC (≥4.0 µg ml^−1^) showed no change (Table S4A, Fig. S2A).

**Table 5.** Combinations of sequence variants with associated AZM MICs

Loci with variants*						No. of isolates per MIC (µg ml^−1^)†						
23 S-C2611	23 S-A2059	*mtrR* mosaic^#^	*mtrR* promoter	MtrD K823	*mtrC* GC del	≤0.5	1	2	4	8	≥16	MLST‡
T	A	TRUE	C	E	2 bp	1	0	0	0	0	0	
T	A	TRUE	C	E	WT	0	0	**2**	**1**	**1**	**2**	9363
T	A	FALSE	A	E	WT	2	0	0	0	**1**	0	9363
T	A	FALSE	A	K	WT	1	0	0	**2**	0	0	1580, 10 932
T	A	FALSE	DEL	K	WT	0	0	0	**1**	**3**	**8**	1579, 8156
C	G	TRUE	C	E	WT	0	0	0	0	0	**2**	9363, 14 101
C	A	TRUE	C	E	2 bp	5	0	0	0	0	0	
C	A	TRUE	C	E	WT	14	87	**73**	**3**	0	**1**	9363, 11 422
C	A	TRUE	C	K	WT	2	1	0	0	0	0	
C	A	FALSE	A	E	2 bp	10	0	0	0	0	0	
C	A	FALSE	A	E	WT	158	19	0	0	0	0	
C	A	FALSE	C	E	WT	2	10	**1**	0	0	0	10 314
C	A	FALSE	DEL	E	WT	4	0	0	0	0	0	
C	A	FALSE	A	NF§	WT	3	0	0	0	0	0	
C	A	FALSE	DEL	NF§	WT	4	0	0	0	0	0	
C	A	FALSE	A	K	2 bp	68	0	0	0	0	0	
C	A	FALSE	C	K	2 bp	1	0	0	0	0	0	
C	A	FALSE	DEL	K	2 bp	30	0	0	0	0	0	
C	A	FALSE	A	K	4 bp	2	0	0	0	0	0	
C	A	FALSE	A	K	WT	902	4	0	0	0	0	
C	A	FALSE	C	K	WT	2	1	0	0	0	0	
C	A	FALSE	DEL	K	WT	268	7	0	0	0	0	
C	A	FALSE	G	K	WT	1	0	0	0	0	0	

*Grey shading indicates the variant compared to unshaded wild-type.

†Elevated MICs: low=2 µg ml^−1^, medium=4–8 µg ml^−1^, high ≥16 µg ml^−1^.

‡Lists the MLST sequence type where isolates with elevated MICs (numbers in bold) with the respective variant profile are most frequently found.

§NF=not found; indicates AMR Profiler and Typing Tool could not make an allele call.

#*mtrR* mosaic indicates whether it is TRUE=*mtrR* genetic sequence is mosaic or FALSE=*mtrR* sequence is not mosaic/closer in identity to CP012026.1 (Table S2).

AZM, azithromycin; MIC, minimum inhibitory concentration; MLST, multilocus sequence typing.

#### Isolates with low-level AZM^em^ (MIC 2.0 µg ml^−1^)

Of the 76 isolates with low-level AZM^em^, 98.7 % (75/76) were phylogenetically located in clade 20 with a combination of STs; 57 % (43/76) were ST9363, 33 % (25/76) ST11422, and 5 % (4/76) ST8134 (Figs 1 and 3, inset Fig. 4). These isolates had a mosaic *mtr* operon, including mosaic *mtrR*, *mtrR* promoter C substitution, and MtrD S821A and K823E, which is capable of elevating AZM MIC (Table 5) [14, 15, 41]. Of those isolates in clade 20, which carried mosaic *mtr* operon (defined as subclade 20.2), 84.9 % (163/192) of isolates had an AZM MIC 1.0–2.0 µg ml^−1^ (chi-square for AZM MIC 1.0–2.0 µg ml^−1^ mosaic *mtr*, χ2(1, *N*=1685) = 1121.574, *P*<0.01) (Table S6), and 99.5 % (191/192) carried MtrD K823E. Subclade 20.2 represented a patient population of 42 % (81/192) MSW and 42 % (81/192) MSM (which represents a statistically higher percent MSM than the overall population, *P* value 4.8 e^−06^) (Table S5).

Overall, 48.4 % (180/372) of clade 20 consisted of subclade 20.1, of which 91.7 % (165/180) had AZM MIC≤0.5 µg ml^−1^. While none in subclade 20.1 contained mosaic *mtrR* (gene and/or promoter), they did contain the MtrD S821A and K823E variants indicative of a mosaic *mtrD* (95.0 %, 171/180). Subclade 20.1 was represented by 59 % (106/180) MSW and 23 % (42/180) MSM, similar to the overall population (Table S5).

In total, 230 isolates in the whole dataset (13.5 %) had AZM MIC≥1 µg ml^−1^. Altogether, 203 of those 230 (88.3 %) had the MtrD K823E variant; 4/203 did not have S821A which often accompanies K823E in a mosaic *mtrD* sequence, therefore it is possible these could be examples of K823E occurring as the result of a SNP versus as part of a mosaic/recombination event. Therefore, 172 of the 203 (84.7 %) also had mosaic *mtrR*, and 185 of the 203 (91.1 %) were in clade 20. Isolates with mosaic elements of the *mtrRCDE* operon (as defined by mosaic *mtrR* and/or MtrD K823E) made up 23.5 % (402/1710) of the 2019 GISP dataset, with 90.3 % (363/402) found in clade 20 (Fig. S2E). The remaining 9.7 % were scattered across the phylogeny among clades 17, 18, 15, 11, 19 and 2, suggesting multiple genetic origins; thus, we sought to investigate the predominant, phylogenetically related, mosaic *mtr* alleles within clade 20.

**Fig. 5.**
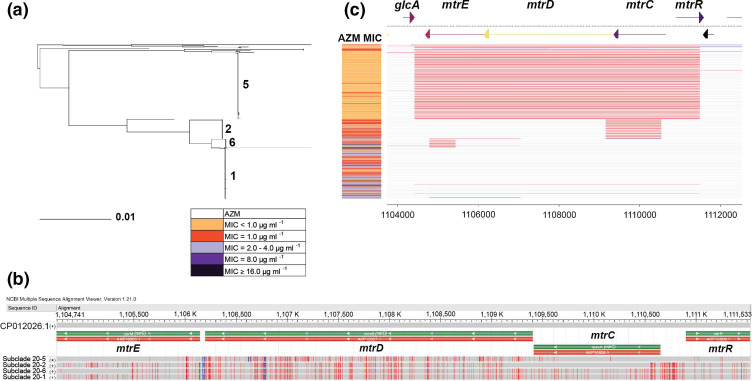
Four prominent clades of mosaic *mtr* operon alleles. (a) Sequences of the *mtrRCDE* operon (6793 bp) from the isolates in clade 20 (*n*=372) were aligned and visualized in a neighbour-joining tree. Fastbaps clustering revealed four main subclades (1, 2, 5 and 6). Azithromycin MICs are indicated by the colours shown in the key. (b) Representative sequences from each subclade were aligned showing the density of variants (SNPs=red bars, insertions/deletions=blue bars) representing the mosaic/recombined areas and where they correspond to on the annotated FA19 (GenBank accession number CP012026.1) sequence (grey regions). Annotations include *Pseudomonas aeruginosa* nomenclature: *oprM* for *mtrE*, *mexB* for *mtrD* and *mexA* for *mtrC*. C. Recombinant regions were called using Gubbins on the 372 sequences from clade 20 and are aligned with the tree in (a). Grey areas represent the most prevalent sequences, while red indicates regions of recombination that are shared by multiple isolates in comparison to the grey. Blue indicates a region of sequence unique to that isolate.

To take a closer look at the different mosaic alleles in clade 20, the *mtr* operon sequences were aligned and four main subclades (1, 2, 5 and 6) were identified (Fig. 5a). Sequences within each subclade were aligned revealing that the majority of sequences (90.3 % for subclade 1, 97.9 % for subclade 2, 86.1 % for subclade 5, and 90.5 % for subclade 6) within each subclade was 100 % identical; the remaining sequences within each subclade had high percent identity (97.87–99.99 %). The representative sequences from subclades 1, 2, 5 and 6 were aligned to the *mtr* operon sequence of FA19 (CP012026.1) (considered wild-type here) to show dense regions of SNPs adjacent to blocks of sequence identical to FA19 (for subclades 5, 2 and 6), indicating different mosaic patterns for each subclade. Subclade 1 (*n*=124) had the entire operon [nucleotide positions 1104741–1111533 (CP012026.1 coordinates)] with a mosaic pattern; subclades 6 (*n*=21) and 2 (*n*=47) showed this same region as mosaic, with the exception of a wild-type (WT) ~690 bp portion of *mtrE* in subclade 6, and a WT ~1440 bp section of *mtrC* and the beginning of *mtrD* in subclade 2. Subclade 5 (*n*=165) had a distinct and shorter mosaic pattern (~3723 bp) compared to the others, which covered only *mtrD* and the neighbouring ends of *mtrC* and *mtrE*; this subclade was most similar to FA19. The mosaic patterns seen in this alignment corroborate with the clustering in the core-genome phylogeny and the associated resistance marker calls (i.e. mosaic *mtrR*, *mtrR* promoter C SNP and MtrD K823E) for the whole-genome sequences (Fig. 4a).

Furthermore, Gubbins analysis was used to look at recombination in clade 20 focusing on the *mtr* operon (Fig. 5). Since subclades 2, 6 and 1 had very similar sequences with almost identical mosaic SNP patterns as seen in the alignment, and cumulatively they totaled more than the number of isolates in subclade 5, the subclade 1 sequence appeared to serve as the reference sequence in the analysis. Thus, the red recombinant regions denoted by Gubbins were highlighted in subclade 5, which does carry a unique mosaic pattern with respect to the other clades. The Gubbins recombinant blocks in subclade 6 (Fig. 5) and the alignment do however come together where there is wild-type sequence (grey block in the alignment) in part of *mtrE* (Fig. 5). Likewise, for subclade 2 a Gubbins recombinant block covers most of *mtrC* and into the beginning of *mtrD* that corresponds to the wild-type sequence (grey section seen in the alignment, 5B), which are identical to the FA19 sequence. The subclade 5 *mtr* operon sequence appears as a Gubbins recombinant block due to most of *mtrR*, the promoter region, *mtrC* and *mtrE* being highly similar to FA19, while *mtrD* has a large number of SNPs with a different mosaic pattern compared to the other subclades (Fig. 5b).

The percentage of isolates with AZM^em^ differed among the subclades. Subclade 5, with only a mosaic *mtrD*, had 95 % of its isolates with AZM MIC <1.0 µg ml^−1^. The other clades (with mosaicity across various regions of *mtrR*, promoter, *mtrCDE*) all had >70 % of isolates with AZM MICs of 1.0–2.0 µg ml^−1^ (Table S7A). blastn queries of the subclade representative *mtr* operon sequences resulted in top hits that were primarily commensal *Neisseria* species (including *N. polysaccharea *, *N. lactamica* and *N. meningitidis *) for subclades 1, 2 and 6, suggesting recombination. For subclade 5, only *N. gonorrhoeae* hits were identified. A blast of *mtrD* only from the subclade representative sequences revealed high percent identity to mosaic *mtrD* alleles (*mtrD* variants A, B and C) characterized in Golparian *et al.*, as well as *N. cinerea* (Table S7A) [42].

#### Variants in *mtr* efflux pump result in AZM susceptibility

Of additional importance, deletions recently described by Ma *et al.* resulting in premature stops in *mtrC* were found in the ‘GC’ dinucleotide hexarepeat region [position 1110299–1110309 of *mtrC* in CP012026.1 (FA19)] [43]. Of 117 sequences, 115 have a two bp deletion, and two have a four bp deletion. All isolates were susceptible to AZM, with MICs ≤0.125 µg ml^−1^. One isolate (GCWGS-11978) with GC deletion had an MIC of 0.125 µg ml^−1^, despite having two copies of the 23S rRNA C2611T mutation, which typically results in elevated AZM MICs. This suggests the early termination of MtrC is leading to a loss of efflux pump activity in these isolates. Seven isolates were also found to have premature stop codons in *mtrD* (Table S7B). Each occurrence was unique with four being caused by nonsynonymous mutations and three resulting from deletions. MICs for all were either 0.015 or 0.03 µg ml^−1^ (Fig. S2E).

The MtrR G45D mutation was only found in isolates that had no mosaic *mtr* alleles (252/1710). AZM MICs for these ranged from 0.008 to 4.0 µg ml^−1^ (average 0.2 µg ml^−1^); the two isolates with MIC 4.0 µg ml^−1^ both had four copies of the C2611T 23S rRNA mutation.

#### Isolates with medium to high AZM^em^ (MIC ≥4.0 µg ml^−1^)

Out of 1710 isolates in the dataset, 25 isolates (1.5 %) had medium to high-level AZM^em^ (MIC ≥4.0 µg ml^−1^), which was associated with 23S rRNA variants (chi-square for AZM MIC ≥4.0 µg ml^−1^, 23S rRNA, χ2(1, *N*=1710) = 1109.0647, *P*<0.01) (Table S6). In total, 19 of the 25 carried C2611T (* Escherichia coli* numbering) 23S rRNA variants, and two others carried A2059G (* E. coli* numbering) 23S rRNA variants (Table 5). Four isolates with elevated MIC to AZM (≥4.0 µg ml^−1^) did not have either of the 23S rRNA mutations (nor the rare mutation at position 2058). Three of them had an MIC of 4.0 µg ml^−1^ and carried mosaic *mtr* operon. One had an MIC of ≥16 µg ml^−1^, carried mosaic *mtr* operon and a rare *mtrR* promoter −35 G variant (ST7363, clade 15).

Ten of the isolates with C2611T were a set of ST1579 isolates tightly clustered within clade 12. All ten carried four copies of C2611T and had AZM MICs from 8.0 to 16 µg ml^−1^. The isolates were collected from three different sentinel sites from February through December 2019. There was not a significant change in percentage of ST1579 from 2018 to 2019.

Ten isolates in clade 20 (seven of them ST9363) had a range of one to four copies of the C2611T variant, and the AZM MICs ranged from 0.125 to ≥16 µg ml^−1^. The remainder of the isolates with C2611T variants had AZM MICs from 0.5 to 8.0 µg ml^−1^ and were in MLST ST1580, ST1893, ST10932 and ST8156 (two isolates) (Fig. S3, Table S8). Isolates with 23S RNA variants with AZM MICs ≤2.0 µg ml^−1^ had only one or two copies of the C2611T mutation. The isolate with the MIC of 0.125 µg ml^−1^ also carried the deletion causing early termination of MtrC. The two isolates which carried A2059G 23S rRNA variants (in two and four copies) had AZM MIC ≥16 µg ml^−1^ and were from clade 20. They were collected from different sentinel sites approximately 4 months apart.

In previous years, a majority of ST7371 carried C2611T 23S rRNA variants [87 %(20/23) in 2017, 57 %(4/7) in 2018]. However, in 2019 only one ST7371 isolate was recorded, and it lacked C2611T. The percentage of ST7371 significantly decreased from 2018 to 2019 (from 0.47 % to 0.06%, *P* value 2.4 e^−02^) (Table 3). Previously, a high percentage of ST1584 also carried C2611T 23S rRNA variants (47 % in 2017 then down to 0 % in 2018) [18, 19]. An overall decrease in ST1584 isolates occurred from 2018 to 2019 (from 3.99 % to 2.63%, *P* value 3.2 e^−02^), and none carried 23S rRNA variants.

### Cefixime and ceftriaxone

Consistent with a low overall percentage of isolates with elevated MICs to CFM and/or CRO (as defined by the cutoffs in Table 1 and aligned with GISP cutoffs of concern, CFM MIC ≥0.25 µg ml^−1^ and CRO MIC ≥0.125 µg ml^−1^) as reported by GISP [7], there were eight sequenced isolates (8/1710, 0.47%) with CFM^em^ and five isolates (5/1710, 0.29%) with CRO^em^. Two isolates were in both categories, one of which (GCWGS-10753) had CFM MIC 1.0 µg ml^−1^ and CRO MIC 1.0 µg ml^−1^.

The isolate with CFM and CRO MICs of 1.0 µg ml^−1^ was in MLST 1901 and clade 12, and had mosaic *penA* allele 60.001, *mtrR* promoter A deletion, and PorB1b G120K, A121D [44]. Two additional isolates with CFM MIC 0.25 µg ml^−1^ grouped in clade 12, with MLST ST1901 and mosaic *penA* 34.001, *mtrR* promoter A deletion, PorB1b G120K and A121D/N variants (Fig. 4c). The remaining isolates with CFM or CRO elevated MICs were phylogenetically diverse and carried various *penA* alleles (Fig. 2, Table S9B).

### Ciprofloxacin

Overall, 35.79 % of all isolates (612/1710) displayed resistance to CIP (defined as having an MIC greater than or equal to the CLSI resistance breakpoint, MIC ≥1.0 µg ml^−1^, CIP^R^). Eight MLST types accounted for 72.7 % (445/612) of all CIP^R^ isolates (Fig. S4A). A subset of CIP^R^ isolates phylogenetically grouped in clades 18 (ST8143, ST13526) and 15 (ST7363) and 11 (ST1588). An additional subset grouped in clades 17 (ST10314, ST7822) and 12 (ST1901, ST1579) (Figs 3 and 4b, c).

CIP resistance was predominantly determined by GyrA S91F variant (chi-square for CIP GyrA, χ2(1, *N*=1710) = 1510.9567, *P*<0.01) (Table S6). However, combinations of variants within GyrA and ParC were identified with the various sequence types (Figs 1 and 4b, c and Table 6). Only 11/612(1.8 %) CIP^R^ did not carry GyrA S91F.

**Table 6.** Combinations of sequence variants with associated CIP MICs

	Loci with variants*					No. of isolates per MIC (µg ml^−1^)†								
GyrA S91	GyrA D95	ParC D86	ParC S87	ParC S88	ParC E91	≤0.25	0.5	1	2	4	8	16	32	MLST‡
F	A	N	S	S	E	2	0	**1**	**35**	**48**	**11**	**10**	0	8143, 9363
F	A	D	N	S	Q	0	0	0	0	**1**	0	0	0	11 956
F	A	D	N	S	K	0	0	0	0	**2**	0	0	0	1588
F	A	D	N	S	E	0	3	**8**	**12**	**5**	0	0	0	1588
F	A	D	R	S	E	5	0	0	**6**	**192**	**92**	**9**	**3**	10314, 13526, 8143,7822
F	A	D	S	S	G	0	0	0	0	1	1	0	0	15 679
F	A	D	S	S	E	8	12	**11**	**4**	0	0	0	0	1583
F	G	N	S	S	E	2	0	0	**7**	**3**	**6**	0	0	7827
F	G	D	I	S	G	0	0	0	0	0	**1**	**1**	**1**	7363
F	G	D	I	S	E	0	0	0	0	0	0	**1**	0	7363
F	G	D	R	S	E	0	0	0	**2**	**26**	**12**	**16**	**1**	1901
F	G	D	S	S	G	0	0	0	**1**	**21**	**31**	**13**	**1**	7363
F	G	D	S	S	E	3	2	0	**1**	**1**	**2**	0	0	7363, 1579, 11648, 10 932
F	N	D	R	P	E	0	0	0	0	0	0	**1**	0	7363
T	D	D	S	S	E	2	0	0	0	0	0	0	0	
S	A	N	S	S	E	2	0	0	0	0	0	0	0	
S	A	D	R	S	E	1	0	0	0	0	0	0	0	
S	G	D	S	S	E	14	0	0	0	0	0	0	0	
S	D	D	N	S	E	1	0	0	0	0	0	0	0	
S	D	D	R	S	E	14	0	0	0	**1**	0	0	0	8143
S	D	D	S	S	G	2	0	0	0	0	0	0	0	
S	D	D	S	S	E	1024	1	**1**	**1**	**6**	**1**	**0**	**1**	9363

*Grey shading indicates the variant compared to unshaded wild-type.

†Resistant MICs: ≥1 µg ml^−1^.

‡Lists the MLST sequence type where CIP resistant isolates (numbers in bold) with the respective variant profile are most frequently found.

CIP, ciprofloxacin; MIC, minimum inhibitory concentration; MLST, multilocus sequence typing.

The number of isolates in ST10314 [97 %(110/113) of which carried CIP^R^] significantly increased in percentage from 3.3 %(49/1479) in 2018 to 6.6 %(113/1710) in 2019 (*P* value 1.9 e^−05^). In addition, a minor subset derived from clade 17, ST9902 [which carried CIP^R^, and the same variants as ST10314 (GyrA S91F, D95A, ParC S87R), NG-STAR ST2148], increased in percentage from 0.2 % in 2018 to 0.7 % in 2019 (*P* value 4.1 e^−02^). No significant increase of percentage of ST8143 or ST13526 of clade 18 was identified.

### Penicillin

In total, 12.98 % (222/1710) of isolates were resistant to penicillin (defined as having an MIC greater than or equal to the CLSI resistance breakpoint, MIC ≥2.0 µg ml^−1^, PEN^R^). PEN^R^ was observed in isolates carrying the *bla*_TEM_ plasmid (chi-square for PEN *bla*_TEM_, χ2(1, *N*=1710) = 1060.7114, *P*<0.01) (Tables 7 and S6). One-half of the PEN^R^ isolates (50.4 %, 112/222) grouped in clade 18 (ST8143 and ST13526), and clade 11 (ST1588) (Fig. 4b). Altogether, 48.6 % (90/185) of clade 18 and 57.5 % (23/40) of clade 11 (ST1588) carried *bla*_TEM_, resulting in PEN MICs ranging from 1.0 to 16 µg ml^−1^ (Fig. S4B). Thus, 100 % (24/24) of clade 1 (ST1583) isolates carried the *bla*_TEM_ plasmid but had a lower range of MICs at 1.0–4.0 µg ml^−1^ (of which only one with an MIC 1.0 µg ml^−1^ was negative for beta-lactamase by laboratory test).

**Table 7.** Combinations of sequence variants with associated PEN MICs

Loci with variants*				No. of isolates per MIC (µg ml^−1^)†								
***bla*_TEM_ present**	**PBP2 (*penA*) D345 insert**	***mtrR* promoter**	**PBP1 (*ponA*) L421**	**≤0.5**	**1**	**2**	**4**	**8**	**16**	**32**	**64**	**MLST‡**
TRUE	TRUE	DEL	P	1	0	0	0	0	0	0	0	
TRUE	TRUE	C	L	1	0	0	0	0	0	0	0	
TRUE	TRUE	A	P	1	2	**4**	**7**	**6**	**3**	**1**	**4**	1588, 8143
TRUE	TRUE	A	L	4	3	**21**	**13**	**5**	**6**	**22**	**65**	8143, 13526, 1583
TRUE	FALSE	A	L	1	0	0	0	0	0	**1**	0	7363
FALSE	TRUE	G	L	1	0	0	0	0	0	0	0	
FALSE	TRUE	DEL	P	171	75	**17**	**2**	0	0	0	0	12093, 1901, 1579
FALSE	TRUE	DEL	L	17	2	**1**	0	0	0	0	0	7827
FALSE	TRUE	C	P	8	4	**1**	0	0	0	0	0	7363
FALSE	TRUE	C	L	120	75	**1**	0	0	0	0	0	11 422
FALSE	TRUE	A	P	117	122	**30**	0	0	0	0	**1**	10 314
FALSE	TRUE	A	L	628	26	**3**	**1**	0	0	0	0	12 905, 8143, 16 161, 1893
FALSE	FALSE	DEL	P	23	9	**3**	**3**	0	0	0	0	1901
FALSE	FALSE	DEL	L	0	1	0	0	0	0	0	0	
FALSE	FALSE	C	L	2	0	0	0	0	0	0	0	
FALSE	FALSE	A	P	1	0	**1**	0	0	0	0	0	7363
FALSE	FALSE	A	L	73	0	0	0	0	0	0	0	

*Grey shading indicates the variant compared to unshaded wild-type.

†Resistant MICs: ≥2 µg ml^−1^.

‡Lists the MLST sequence type where PEN-resistant isolates (numbers in bold) with the respective variant profile are most frequently found.

MIC, minimum inhibitory concentration; MLST, multilocus sequence typing; PEN, penicillin.

PEN^R^ in the range of MIC 2.0–4.0 µg ml^−1^ and in the absence of *bla*_TEM_ was seen across clades 12 and 17. The isolates carried combinations of other variants known to be associated with resistance: PBP1 L421P, *penA* D345 insertion, and in some the *mtrR* promoter A deletion and PorB1b G120 D/K and A121 D/N variants) (Table 7).

### Tetracycline

In total, 27.6 % (472/1710) of isolates were tetracycline resistant (defined as having an MIC greater than or equal to the CLSI resistance breakpoint, MIC ≥2.0 µg ml^−1^, TET^R^). 64.6 % (305/472) had TET MIC 2.0–4.0 µg ml^−1^, while 35.3 % (167/472) had TET MIC ≥8.0 µg ml^−1^.

The TET^R^ isolates with MIC 8.0–64 µg ml^−1^ carried the *tetM* plasmid (chi-square for TET^R^ MIC ≥8.0 µg ml^−1^, *tetM*, χ2 (1, *N*=1710) = 1676.0545, *P*<0.01) (Table S6) and were predominately in clade 7 [ST1599 (69/80)], clade 1 [ST1583 (24/24)], and clade 8 [ST10932 and ST13533 (21/21)]. A small subset of clade 14 [ST1893 (12/54)] and clade 11 [ST1588 (11/40)] also carried *tetM* (Figs 1 and S4C, Table 8).

**Table 8.** Combinations of sequence variants with associated TET MICs

Loci with variants*					No. of isolates per MIC (µg ml^−1^)†								
*tetM* present	RpsJ V57	*mtrR* promoter	PorB aa120	PorB aa121	≤0.5	1	2	4	8	16	32	64	MLST‡
TRUE	M	DEL	GN	AGSV	0	0	0	0	0	0	**4#**	0	§
TRUE	M	A	DK	DN	0	0	0	0	0	0	**1**	**3**	1583, 16189, 13 413
TRUE	M	A	DK	AGSV	0	0	0	0	0	**1#**	**5#**	**1**	§
TRUE	M	A	GN	AGSV	1	0	0	0	0	**11#**	**119**	**9**	1599, 1583
TRUE	M	A	GN	DN	0	0	0	0	0	0	**1**	0	10 932
TRUE	M	A	n/a	n/a	0	0	0	0	0	0	**3**	**1**	12462, 1599
TRUE	V	C	GN	AGSV	0	0	0	0	0	0	**1**	0	9363
TRUE	V	A	GN	AGSV	0	0	0	0	**1**	**2**	**2**	0	10932, 11 184
FALSE	M	DEL	DK	DN	4	17	**60**	**15**	0	0	0	0	7363, 1901
FALSE	M	DEL	DK	AGSV	0	1**#**	**1#**	0	0	0	0	0	8143
FALSE	M	DEL	GN	DN	3	3	**1**	**1**	0	0	0	0	1901
FALSE	M	DEL	GN	AGSV	24**#**	79**#**	**5**	0	0	0	0	0	§
FALSE	M	DEL	n/a	n/a	0	7	**2**	0	0	0	**1**	0	13536, 7827
FALSE	M	C	DK	DN	2	5	**87**	**20**	0	0	0	0	9363, 11 422
FALSE	M	C	DK	AGSV	0	2**#**	**1#**	0	0	0	0	0	9363
FALSE	M	C	GN	AGSV	14**#**	64**#**	**10**	0	0	0	0	0	9363, 8134
FALSE	M	C	GN	DN	0	0	**1**	0	0	0	0	0	9363
FALSE	M	C	n/a	n/a	0	0	**2**	0	0	0	0	0	9363, 11 422
FALSE	M	A	DK	AGSV	41**#**	62**#**	**9#**	**1**	0	0	0	0	8143, 1893
FALSE	M	A	DK	DN	2	20	**15**	**2**	0	0	0	0	7822, 10 314
FALSE	M	A	GN	AGSV	189**#**	439**#**	**67#**	**1**	0	0	0	0	9363, 10314, 13413, 1901, 8143
FALSE	M	A	GN	DN	0	4	**2**	0	0	0	0	0	1901, 7363
FALSE	M	A	n/a	n/a	3	7	0	0	0	0	0	0	
FALSE	V	G	GN	AGSV	1	0	0	0	0	0	0	0	
FALSE	V	DEL	DK	DN	29	0	**1**	0	0	0	0	0	10 931
FALSE	V	DEL	DK	AGSV	1	0	0	0	0	0	0	0	
FALSE	V	DEL	GN	AGSV	66	0	0	0	0	0	0	0	
FALSE	V	C	GN	AGSV	3	0	0	0	0	0	0	0	
FALSE	V	A	DK	DN	4	0	**1**	0	0	0	0	0	9362
FALSE	V	A	DK	AGSV	2	0	0	0	0	0	0	0	
FALSE	V	A	GN	AGSV	137	2	0	0	0	**1**	0	0	6962

*Grey shading indicates a variant potentially contributing to changes in MIC.

†Resistant MICs: ≥2 µg ml^−1^.

‡Lists the MLST sequence type where TET-resistant isolates (numbers in bold) with the respective variant profile are most frequently found.

§No predominant MLST, all resistant isolates in different STs.

#Includes one or more isolates with porB1a alleles. See Table S10 for more on porB1a.

MIC, minimum inhibitory concentration; MLST, multilocus sequence typing; TET, tetracycline.

The TET^R^ isolates with MIC 2.0–4.0 µg ml^−1^, all of which did not carry *tetM*, carried combinations of other variants known to contribute to resistance (RpsJ V57M, *mtrR* promoter A deletion or C, and PorB1b G120 D/K and A121 D/N) [5] (Table 8). Overall, 99 % of these isolates (303/305) carried the RpsJ V57M variant. 60 % (183/305) of these isolates had an *mtrR* promoter mutation along with PorB1b G120D/K (and A121D/N); this subset was primarily found in clade 20 (105/182) and clade 15 (40/182).

## Discussion

This annual analysis of 1710 GC isolates from GISP sentinel surveillance sites provides a detailed report of the strain diversity and characterization of common genetic markers associated with reduced susceptibilities to antibiotics of concern present in the USA in 2019. Based on this established sentinel surveillance programme, the 2019 dataset and previous 2018 WGS dataset allowed a side-by-side comparison to monitor the increase or decrease of strain presence and the identification and change in frequency of antimicrobial-resistant markers between 2 consecutive years [19]. Continued analyses in future years will allow us to understand better the trends in strain diversity over time.

Limitations of this study include the collection of only male urethral isolates; GISP collects from 32 sentinel sites, which may not be representative of all *N. gonorrhoea* in the USA; and not all GISP sites provided 100 % of the first five GISP isolates for the year, which may or may not have biassed our findings. The WGS data set, however, was demographically representative of the total GISP population in 2019 (Table 2).

Overall, a majority of the MLST STs present and the percentages of STs in 2019 were similar to previous years. Noteworthy differences included the expansion of several sequence types (e.g. ST10314) and the loss of others (ST1584 and ST7371) (Table 3). This dataset also allowed the recording of increased numbers of novel sequence types, presumably due to expansion of existing strains (e.g. ST9902 from ST10314).

Established patterns of plasmids being the source of PEN and TET MICs ≥4.0 µg ml^−1^ continued to hold true in 2019. Additionally, chromosomally encoded mutations in *rpsJ*, *porB*, the *mtrR* promoter, or *penA* appear to have accumulated to build MICs 2.0 µg ml^−1^ for TET and PEN as previously observed [5, 10]. Elevated MICs (resulting in resistance or reduced susceptibility) to more than one antibiotic was distinctly apparent among several clades in the 2019 phylogenetic tree. Isolates in clade 20 featured AZM^em^, TET^R^ and CIP^R^. Clade 15 contained many CIP^R^ and TET^R^ isolates, clade 18 includes CIP^R^ and PEN^R^, and clade 12 has CIP^R^ isolates co-occurring with either PEN^R^, TET^R^, or AZM^em^ (Fig. 3).

Nationally, the overall percentage of CIP^R^ in the USA increased 4.19 % (from 31.2–35.4 %) from 2018 to 2019 according to GISP, even though CIP was not a recommended treatment for gonorrhoea in the USA [7]. In particular, clade 17 (ST10314 and ST9902) showed an increase in percentage that accounted for 96 % of the total CIP^R^ increase in 2019 (Table 3). Comparison of the GISP annual summaries showed an overall 2.15 % increase of TET^R^ isolates from 2018 to 2019 (25.62 %–27.77%), which was accounted for by the increase of isolates with chromosomally based TET^R^ (MIC of 2.0–4.0 µg ml^−1^) [7, 45].

The continued expansion of clade 20 (with low-level AZM^em^ and mosaic *mtr* operon) from 2018 to 2019 was responsible for the overall reported annual percent increase of isolates with elevated AZM MICs (all at 2.0 µg ml^−1^ and defined as nonsusceptible by CLSI breakpoints) within the USA [7, 20]. Across the major MLST sequence types in clade 20, none showed a significant decrease in percentage, and several, including ST8134 (first identified in the USA in 2018) and ST11982, showed expansion (Table 3). Nearly half of the MLST sequence types in clade 20 (9/20, 45%) were represented by two or fewer isolates, with many previously unidentified STs. This suggests genetic variability across the sequence types within this group.

GC as a species is well known to readily undergo homologous recombination with other *Neisseria* species, often resulting in mosaic alleles. Previous reports presented various mosaic *mtr* alleles that were likely created from recombination with *N. meningitidis *, *N. lactamica *, etc [14, 15]. Analysis of the prominent *mtr* operon alleles in clade 20 indicated recombination with other *Neisseria* species (such as the aforementioned as well as *N. polysaccharea *) based on alignment with wild-type strain FA19, the mosaic variant calls obtained from genomic AMR profiling, Gubbins calls of recombination blocks, and blast results to *Neisseria* spp. genome database. The different recombination patterns among the subclades (*mtrR*/ promoter/ *mtrCDE* versus *mtrR*/ promoter/ *mtrDE* only) corresponded with different distributions of AZM susceptibility (e.g. ratio of isolates with MIC 1.0–2.0 µg ml^−1^). Further in-depth study across multiple years may provide a greater understanding of the frequency, locations and origins of recombination events within this locus, and how this associates with success and expansion of a single strain type [46]. Extended analysis of mosaic *mtr* alleles beyond clade 20 would also be an important factor for further study of recombination of the *mtrR-CDE* operon. Additionally, continued examination will increase understanding of the effects of the various mosaic *mtr* alleles on antimicrobial resistance and fitness. MtrD in particular, being the component of the efflux pump, which specifies what substrates are pumped out, may present new variants that enhance substrate binding or serve as the basis for accumulation of mutations in other loci that increase antimicrobial resistance [41, 47].

Although the GISP 2019 report documents an overall increase in the proportion of isolates with elevated MICs to AZM from 2018–2019, the report stated a decrease in the proportion of AZM MIC ≥4.0 µg ml^−1^ from 2018 (1.40 %) to 2019 (0.95 %); in comparison to a slight increase in MIC 2.0 µg ml^−1^ from 3.16–4.18 % from 2018 to 2019 [7]. This decrease in the overall percent of isolates with AZM MIC 4.0–16 µg ml^−1^ was associated with a loss of isolates with 23S rRNA variants containing all four copies. In 2019, 23S rRNA variants were common in ST1579 of clade 12(10/11 isolates). Otherwise, and as seen in 2018, the 23S rRNA variants were in single isolates distributed across the phylogenetic tree. Significant expansion was not seen in these strain types; in fact, a decrease was seen in the previously prevalent ST7371 and ST1584. Currently, in 2019, no isolate in ST7371 (0/1) nor ST1584 (0/46) carries 23S rRNA variants. The inability to maintain these variants may indicate a fitness cost is associated with their carriage; however, this is contradictory to an *in vivo* study which suggests the A2059G variant may confer a fitness advantage [48].

In alignment with the annual GISP 2019 report [7], there were only a few isolates in this dataset with elevated MICs to CFM or CRO. The isolates with elevated MICs were phylogenetically diverse, from multiple STs, and carried various *penA* alleles (10.001, 27.002, 34.001, 60.001, 67.001; Table S9). The first isolate in the USA with CRO MIC 1.0 µg ml^−1^ and *penA* 60.001 was identified in Las Vegas in 2019 through the GISP programme and is included in this dataset [44]. Even with additional monitoring in Las Vegas at that time (including culture-independent molecular testing for mosaic *penA* 60.001 of 257 remnant NAAT specimens from southern Nevada), no other isolate which carried the *penA* 60.001 or which had a CRO MIC 1.0 µg ml^−1^) was identified [44]. The identification of the isolate with CRO MIC 1.0 µg ml^−1^ in Las Vegas, of course, reaffirmed CDC of the necessity for [1] continued national culture-based surveillance and antibiotic susceptibility testing [2], a documented protocol for rapid, coordinated public health response and surveillance at a local jurisdiction in response to identification of an isolate of concern, and [3] continued development of rapid, molecular assays for GC identification, strain typing and detection of AR markers in light of GC’s rapid evolution.

In conclusion, this dataset of 1710 surveillance-based genomic sequences from GISP provides high-resolution details for the monitoring of strain diversity, strain proportion and antimicrobial resistance determinants in the USA in 2019. Genomic data in alignment with phenotypic and patient demographic data can support the tracking of strain trends and in time can be used to identify and track outbreaks of antibiotic resistant GC. Continued assessment of the sequence types and antimicrobial-resistant determinants present in the USA can direct and facilitate the development and refinement of clinical diagnostic assays for strain specific-GC detection and for antimicrobial susceptibility for targeted therapies. This snapshot of genetic profiles of GC within the USA in 2019 provides data for the understanding of the introduction, persistence and expansion of strains (in particular, strains with elevated MIC or resistance to antibiotics), which will be invaluable in the development of public health responses to reduce the propagation of GC and antimicrobial-resistant strains of GC.

## Supplementary Data

Supplementary material 1

Supplementary material 2
