# Supplementary material for: Genomic analysis of 1710 surveillance-based Neisseria gonorrhoeae isolates from the USA in 2019 identifies predominant strain types and chromosomal antimicrobial-resistance determinants
Source: Microb Genom. 2023 May 12;9(5):mgen001006. doi: 10.1099/mgen.0.001006 (PMC10272886; doi:10.1099/mgen.0.001006)
Supplement: Supplementary material 1 [file mgen-9-1006-s001.pdf]

Supplemental Materials

Microbial Genomics.

Genomic analysis of 1710 surveillance-based *Neisseria gonorrhoeae* isolates from the U.S. in 2019 identifies predominant strain types and chromosomal antimicrobial resistant determinants

Jennifer L Reimche, PhD *†, Arvon Clemons II, M.S. *†, Vasanta L Chivukula, PhD§†, Sandeep J Joseph, PhD, MPH*, Matthew W Schmerer, PhD*, Cau D Pham, PhD*, Karen Schlanger, PhD*, Sancta B St Cyr, MD*, Ellen N Kersh, PhD*, Kim M Gernert, PhD*
Antimicrobial- Resistant *Neisseria gonorrhoeae* Working Group

*Division of STD Prevention, National Center for HIV/AIDS, Viral Hepatitis, STD and TB Prevention, Centers for Disease Control and Prevention, Atlanta, GA;
§ Division of Preparedness and Emerging Infections, National Center for Emerging and Zoonotic Infectious Diseases, Centers for Disease Control and Prevention, Atlanta, GA; and
†Oak Ridge Institute for Science and Education Research Participation and Fellowship Program, Oak Ridge, TN

Antimicrobial Resistant *Neisseria gonorrhoeae* Working Group: Sopheay Hun, MBA, Chi Hua, BS, Ryan Ruiz, MS (Antibiotic Resistance Laboratory Network [AR Lab Network], Washington State Department of Health, WA); Olusegun O Soge, PhD (Department of Global Health and Medicine, University of Washington, Seattle, WA); Catherine Dominguez, PhD, Jillian Loomis, BS, Ami Patel, PhD, David Torpey, PhD (AR Lab Network, Maryland Department of Health, MD); Jenny Zhang, MD, Tamara Baldwin, BS, Chun Wang, MS, John Leavitt, PhD (AR Lab Network, Texas Department of State Health Services, TX); Christina Moore, BS (AR Lab Network, Tennessee Department of Health, TN); Erin Young, Jenni Wagner, Kelly Oakeson (Utah Public Health Laboratory) ; Samera Sharpe, BS; Alesia Harvey, BS; Niketta Womack, BS; Emily Learner, PhD, MPH; Kerry Mauk, MSPH; Kristen Kreisel, PhD; Hillard S Weinstock, MD, PhD (Centers for Disease Control and Prevention, National Center for HIV/AIDS, Viral Hepatitis, STD and TB Prevention, Division of STD Prevention, Atlanta, GA).

Table of Contents

- Methods
- Supplemental Table 1. Bioinformatic Methods and References
- Supplemental Table 2. AMR Profiler. Genomic and Protein Variants.
- Supplemental Figure 1A. Distribution of *Neisseria gonorrhoeae* Multi Antigen Sequence Type (ngMAST) of the first 5 isolates
- Supplemental Figure 1B. Distribution of *Neisseria gonorrhoeae* Sequence Typing for Antimicrobial Resistance (NGSTAR) of the first 5 isolates.

- Supplemental Figure 2A. Maximum likelihood core-genome SNP phylogenetic reconstruction of the merged 2018 (n=1479) and 2019 (n=1710) GISP datasets from the United States.
- Supplemental Figures 2B, C, and D. Close-up of clades 20 (B), 17 and 12 (C), and 18 (D) from maximum likelihood core-genome SNP phylogenetic reconstruction of the merged 2018 (n=1479) and 2019 (n=1710) GISP datasets.
- Supplemental Figure 2E. Maximum likelihood core-genome SNP phylogenetic reconstruction of 1710 GISP first 5 isolates from the United States in 2019.
- Supplemental Table 3. Distribution of specimens by MLST and comparison between 2018 – 2019.
- Supplemental Table 4A. Distribution of specimens by MIC to AZM and comparison between 2018 – 2019.
- Supplemental Table 4B. Distribution of specimens by MIC to PEN and comparison between 2018 – 2019.
- Supplemental Table 4C. Distribution of specimens by MIC to TET and comparison between 2018 – 2019.
- Supplemental Table 5. Distribution of isolates per clade based on sex of sex partner.
- Supplemental Table 6. Chi Square for Antibiotics with elevated MICs and the associated genomic variants.
- Supplemental Table 7A. Characteristics of Mosaic *mtr* operon subclades
- Supplemental Table 7B. Seven sequences had NF calls for mtrD from AMR Profiler.
- Supplemental Table 8. Isolates carrying 23S rRNA C2611T variants in fewer than 4 copies
- Supplemental Figure 3. Count of isolates per MLST which carried 23S rRNA variants in fewer than 4 copies.
- Supplemental Table 9. Isolates with CFM^{em} or CRO^{em} were phylogenetically diverse and carried various *penA* alleles.
- Supplemental Figure 4A. Multilocus sequence type distribution of the 1710 GISP 1st 5 isolates from 2019 in the United States, by CIP resistance.
- Supplemental Figure 4B. Multilocus sequence type distribution of the 1710 GISP 1st 5 isolates from 2019 in the United States, by PEN resistance.
- Supplemental Figure 4C. Multilocus sequence type distribution of the 1710 GISP 1st 5 isolates from 2019 in the United States, by TET resistance.
- References

Supplemental Table 1. Bioinformatic Methods and References.

Method	Reference
FastQC 0.11.5	http://www.bioinformatics.babraham.ac.uk/projects/fastqc/
Kraken 0.10.5	https://ccb.jhu.edu/software/kraken/ (1)
StringMLST 0.3.6	http://jordan.biology.gatech.edu/page/software/stringmlst/ (2)
Cutadapt 1.8.3, 1.16	https://cutadapt.readthedocs.io/en/stable/ (3)
SPAdes Genome Assembler 3.9.0	https://cab.spbu.ru/software/spades/ (4)
Quast 4.3	http://quast.sourceforge.net/ (5)
AMR-Profiler and Typing Tool 2.9.2-dev	(6)
breseq v. 0.35.0	https://barricklab.org/twiki/bin/view/Lab/ToolsBacterialGenomeResequencing (7)
SNIPPY 4.3.8	https://github.com/tseemann/snippy
bwa/0.7.12, samtools/1.3.1, freebayes/1.0.2	(8, 9) https://github.com/freebayes/freebayes
NGMASTER 0.4	https://github.com/MDU-PHL/ngmaster (10)
pyngSTar	https://github.com/leosanbu/pyngSTar (11)
ParSNP (Harvest 1.2)	https://harvest.readthedocs.io/en/latest/content/parsnp.html (12)
Gubbins 2.3.1	https://github.com/nickjcroucher/gubbins (13)
RaxML 8.2.9 GTRCAT substitution, 1000 bootstrap	https://cme.h-its.org/exelixis/web/software/raxml/ (14)
Fastbaps	https://github.com/gtonkinhill/fastbaps (15)
Interactive Tree of Life (iTOL)	https://itol.embl.de (16)
ggtree	https://github.com/YuLab-SMU/ggtree (17)
BLASTn (ncbi-blast-2.9.0+)	https://blast.ncbi.nlm.nih.gov/Blast.cgi
Conversion of BLASTn output to fasta file	https://github.com/gradlab/mtrC-GWAS/blob/master/mtrC-GWAS-notebook.ipynb (18)
MAFFT 7.471	https://mafft.cbrc.jp/alignment/software/ (19)

CLC Genomic Workbench 20.0.2	https://digitalinsights.qiagen.com/
NCBI Multiple Sequence Alignment Viewer 1.21.0	https://www.ncbi.nlm.nih.gov/tools/msaviewer/
R (v.4.0.4 “Lost Library Book”) (2021-02-15)<br>RStudio (v. 1.3.1073)	https://cloud.r-project.org
exact2x2 R	https://cran.r-project.org/web/packages/exact2x2/index.html

Supplemental Table 1B. Required Assembly Statistics, Scoring criteria.

Quality Metric	Pass
Expected coverage	≥40x estimated coverage
Q30 (%)*	≥75 (v2); ≥70 (v3)
Read depth of coverage	≥10x read depth across gene loci
Number of contigs	≤200
N50	>20,000
Final genome contig size	2.1 – 2.4 Mb
Percent genome aligned	85 – 100% genome
Percent GC	52.18 – 52.94
% Ng reads out of all *Neisseria species*	≥85%

*Cutoffs suggested by Illumina dependent on whether MiSeq v2 or v3 chemistries are used for sequencing

Supplemental Table 2. AMR Profiler - Genomic and Protein Variants

	**Accession Number: sequence identifier**	**Accession Number: sequence identifier**
**Genomic Variants**	***Nucleotide***	**Amino Acid**
2611C>T	X67293.1: r.2599C>T	
2059A>G	X67293.1: r.2047A>G	
mtr promoter (mtrR and mtrCDE)		
delA	NZ_CP012026.1: g.1110846del	
A>C	NZ_CP012026.1: g.1110846A>C	
mtrR -35A	NZ_CP012026.1: g.1110837G>A	
mtrR premature stop *	NZ_CP012026.1: g. (1110901_?_1111533)del	(AKP10809.1) p.(1_?_210)del
mtrC GC deletion 2 bp (GC), 4 pb (GCGC)	NZ_CP012026.1: g. (1110299-1110309)delGC	(AKP10808.1) p.(341-342)del
**Protein variants**		
MtrR Ala39Thr	NZ_CP012026.1: g.1111015G>A	(AKP10809.1) p.(Ala39Thr)
MtrR Gly45Asp	NZ_CP012026.1: g.1111034G>A	(AKP10809.1) p.(Gly45Asp)
MtrR His105Tyr	NZ_CP012026.1: g.1111213C>T	(AKP10809.1) p.(His105Tyr)
MtrD Ser821Ala	NZ_CP012026.1: g.1106940A>C	(AKP10807.1) p.(Ser821Ala)
MtrD Lys823Glu	NZ_CP012026.1: g.1106934T>C	(AKP10807.1) p.(Lys823Glu)
PorB Gly120Lys	NZ_CP012026.1:g.1598401GGC>AAG g.1598401GGC>AAG	(AKP11294.1) p.(Gly120Asp)
PorB Gly120Asp	NZ CP012026.1: g.1598402G>A	(AKP11294.1) p.(Gly120Lys)
PorB Gly121Asp	NZ_CP012026.1: g.1598404GGC>GAC	(AKP11294.1) p.(Gly121Asp)
PorB Gly121Asn	NZ_CP012026.1: g.1598404GGC>AAC	(AKP11294.1) p.(Gly121Asn)
PBP1 Leu421Pro	NZ_CP012026.1: g.2080172T>C	(AKP11771.1) p.(Leu421Pro)
PBP2 Ala311Val	NZ CP012026.1: g. 1,302,238 G>A	(AKP10994.1) p.(Ala311Val)
PBP2 Asp345 insertion	NZ_CP012026.1: g.1302135+TCG	(AKP10994.1) p.(Asp345ins)
PBP2 Thr483Ser	NZ CP012026.1: g.1301722C>G	(AKP10994.1) p.(Thr483Ser)
PBP2 Ala501Thr	NZ_CP012026.1: g.1301669C>T	(AKP10994.1) p.(Ala501Thr)
PBP2 Ala501Val	NZ_CP012026.1: g.1301668G>A	(AKP10994.1) p.(Ala501Val)
GyrA Ser91Phe	NZ_CP012026.1: g.359891G>A	(AKP10068.1) p.(Ser91Phe)
GyrA Asp95Ala	NZ_CP012026.1: g.359879T>G	(AKP10068.1) p.(Asp95Ala)
GyrA Asp95Gly	NZ_CP012026.1: g.359879T>C	(AKP10068.1) p.(Asp95Gly)
GyrA Asp95Asn	NZ_CP012026.1: g.359880C>T	(AKP10068.1) p.(Asp95Asn)
ParC Asp86Asn	NZ_CP012026.1:g.993818G>A	(AKP10706.1) p.(Asp86Asn)
ParC Ser87Arg	NZ_CP012026.1: g.993821A>C	(AKP10706.1) p.(Ser87Arg)
ParC Ser87Asn	NZ_CP012026.1: g.993822G>A	(AKP10706.1) p.(Ser87Asn)
ParC Ser87Ile	NZ_CP012026.1: g.993822G>T	(AKP10706.1) p.(Ser87Ile)
ParC Ser88Pro	NZ_CP012026.1:g.993824T>C	(AKP10706.1) p.(Ser88Pro)
ParC Glu91Gly	NZ_CP012026.1:g.993834A>G	(AKP10706.1) p.(Glu91Gly)
ParC Glu91Lys	NZ_CP012026.1:g.993833G>A	(AKP10706.1) p.(Glu91Lys)
ParC Glu91Gln	NZ_CP012026.1:g.993833G>C	(AKP10706.1) p.(Glu91Gln)

RpsJ Val57Met V57M	NZ_CP012026.1:g.1616961C>T	(AKP11325.1) p.(Val57Met)
AcnB Gln57Lys	NZ_CP012026.1:g.964538C>A	(AKP10680.1) p.(Gln57Lys)
**Genomic Mosaicity**		
mtrR mosaic†	KT954125.1: c.(1–797)	
penA mosaic‡	NZ_CP012026.1: c.(1301424-1303169)	

* MtrR premature stop (AKP10809.1) p.(1_?_210)del : The complete nucleotide sequence *mtrR* was scanned for a stop codon or deletion in any position. If a stop codon or deletion was found in any position, it was assigned as a premature stop.
† *mtrR* mosaic KT954125.1: c.1–797 : The complete nucleotide sequence of gene *mtrR* was aligned to the reference to calculate percent similarity and determine mosaicity.
‡ *penA* mosaic NZ_CP012026.1: c.1301424-1303169 : The complete nucleotide sequence of gene *penA* was blasted against the PubMLST database *penA* locus (NEIS1753) to identify *penA* Type and NG STAR *penA* allele. https://pubmlst.org/neisseria/

Supplemental Figure 1A. Distribution of *Neisseria gonorrhoeae* Multi Antigen Sequence Type (ngMAST) of the first 5 isolates per month per sentinel site. Only the STs with at least 6 isolates are represented.

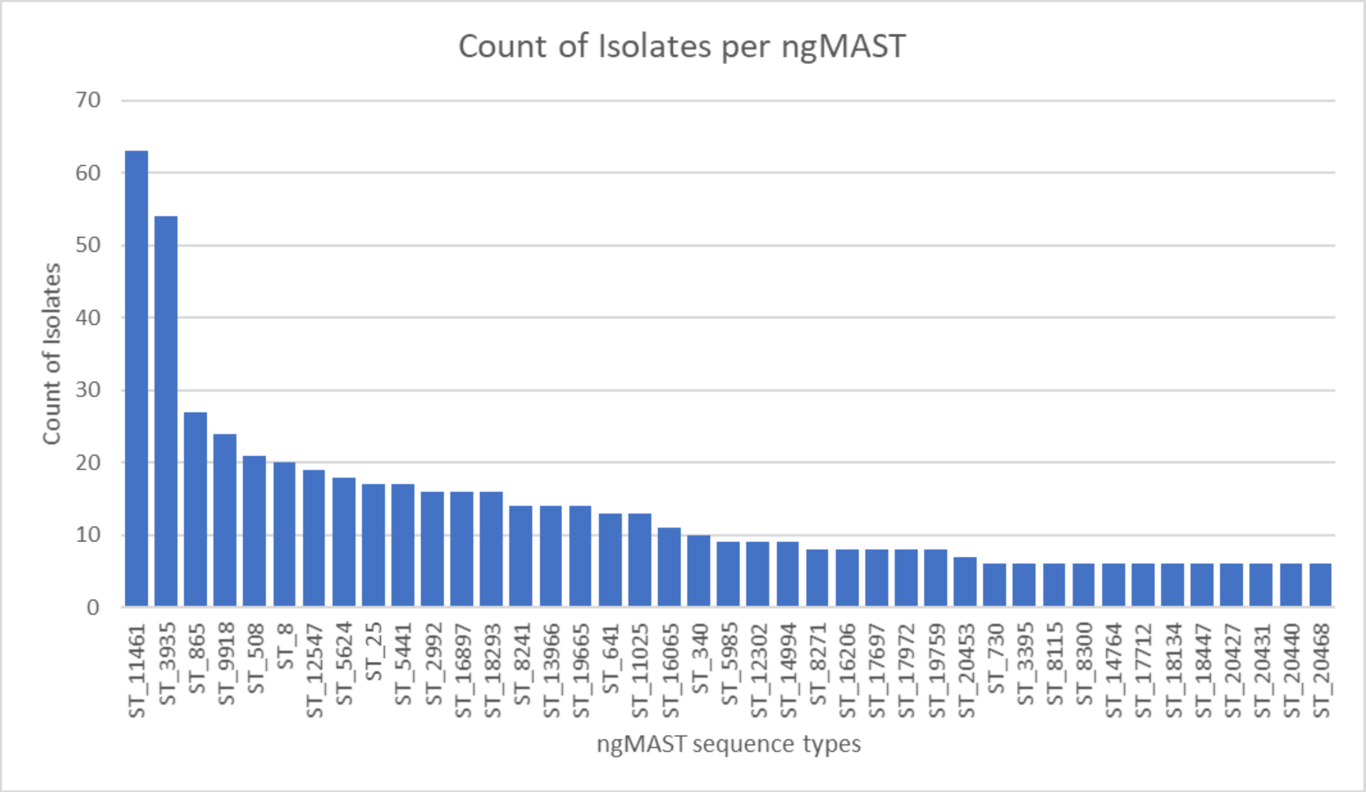

Supplemental Figure 1B. Distribution of *Neisseria gonorrhoeae* Sequence Typing for Antimicrobial Resistance (NG-STAR) of the first five isolates per month per sentinel site. Only the STs with at least 6 isolates are represented.

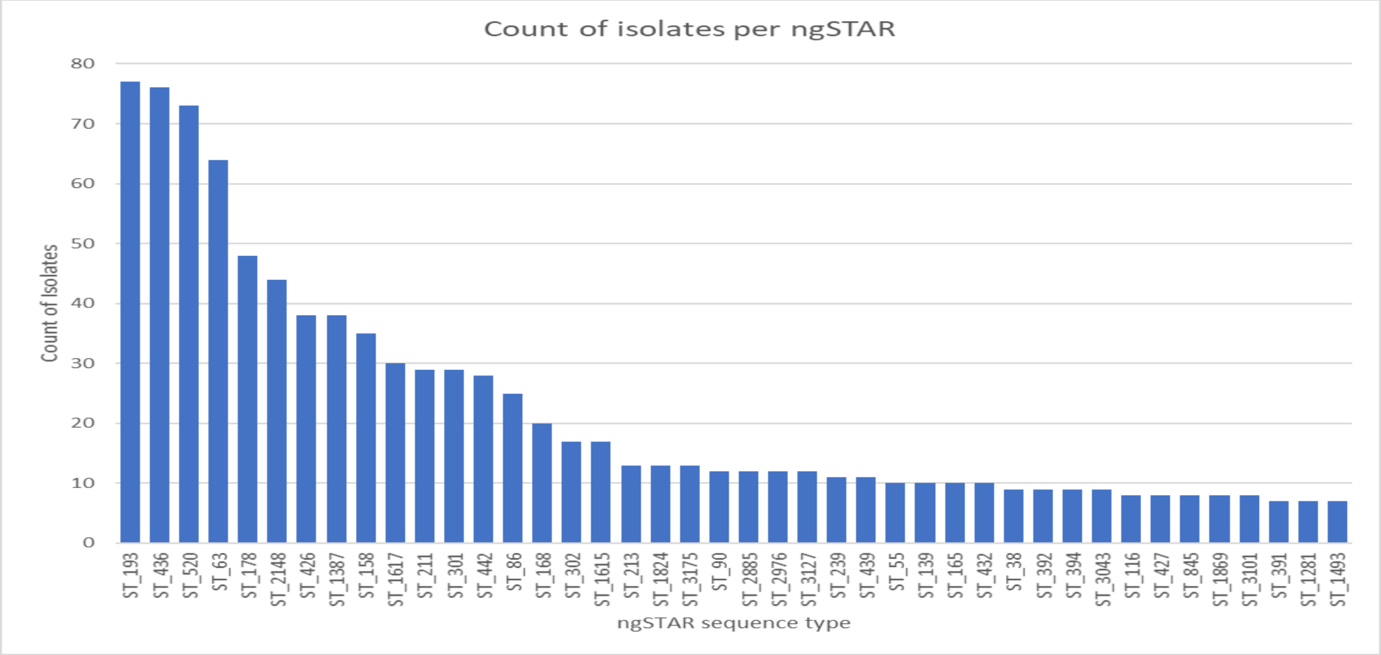

Supplemental Figure 2A. Maximum likelihood core-genome SNP phylogenetic reconstructiont of the merged 2018 (n=1479) and 2019 (n=1710) GISP datasets from the United States.

The clades, as defined by fastbaps, are identified by the inner ring (Clade) and are colored and numbered according the 2019 numbering scheme. Isolates which were not grouped into a predominant phylogenetic clade are uncolored (white). The rings (inner to outer) provide assignments of the following for 2018 (rings 2-7) and 2019 (rings 8-13): MLST, CIP MIC, GyrA S91F, AZM MIC, mosaic *mtrR*, MtrD K823E. The outermost ring shows sex of sex partner. MLST STs are shown (with the color key for Supplemental Figure 2E, listed in approximate order of appearance, with the exception of ST8134 (gold) and ST11982 (red)) and MLST STs ("MLST Label" in right-hand column). MLSTs with low representation are uncolored (white). Isolate susceptibility profiles are shown for CIP and AZM and are colored according to MIC (susceptible (gold), elevated MIC (shades of purple)). The variants are represented as wild-type (gold) or mutant (orange, or light to dark purple). Sex of sex partner is represented as pink for men who have sex with women, dark green for men who have sex with men, light green for men who have sex with men and women, grey for unknown.

A.

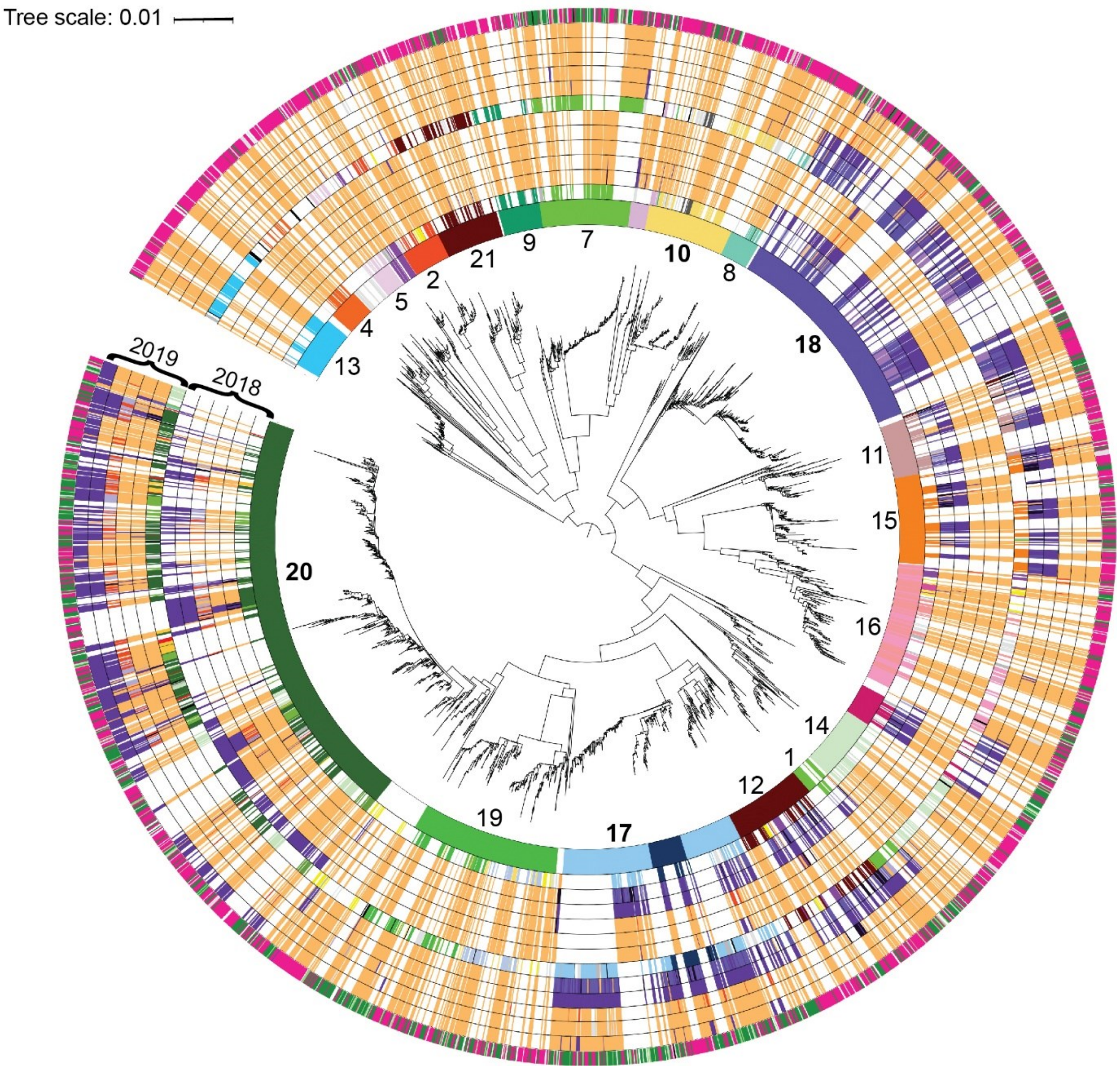

Supplemental Figures 2 B, C, D. Close-up of clades 20 (B), 17 and 12 (C), and 18 (D) from maximum likelihood core-genome SNP phylogenetic reconstruction of the merged 2018 (n=1479) and 2019 (n=1710) GISP datasets.

Antibiotic susceptibility profiles and genetic variant profiles are specified for (B) clade 20 (372/1710 isolates), (C) clade 17 (189/1710 isolates) and clade 12 (61/1710), and (C) clade 18 (185/1710 isolates). Isolate susceptibility profiles are shown for CIP and AZM, and are colored according to MIC (susceptible (gold), elevated MIC (shades of purple)). The variants (GyrA S91F, mosaic *mtrR*, MtrD K823E) are represented as wild-type (gold) or mutant (orange, or light to dark purple). Sex of sex partner is represented as pink for men who have sex with women only, dark green for men who have sex with men only, light green for men who have sex with men and women, and grey for unknown.

B.

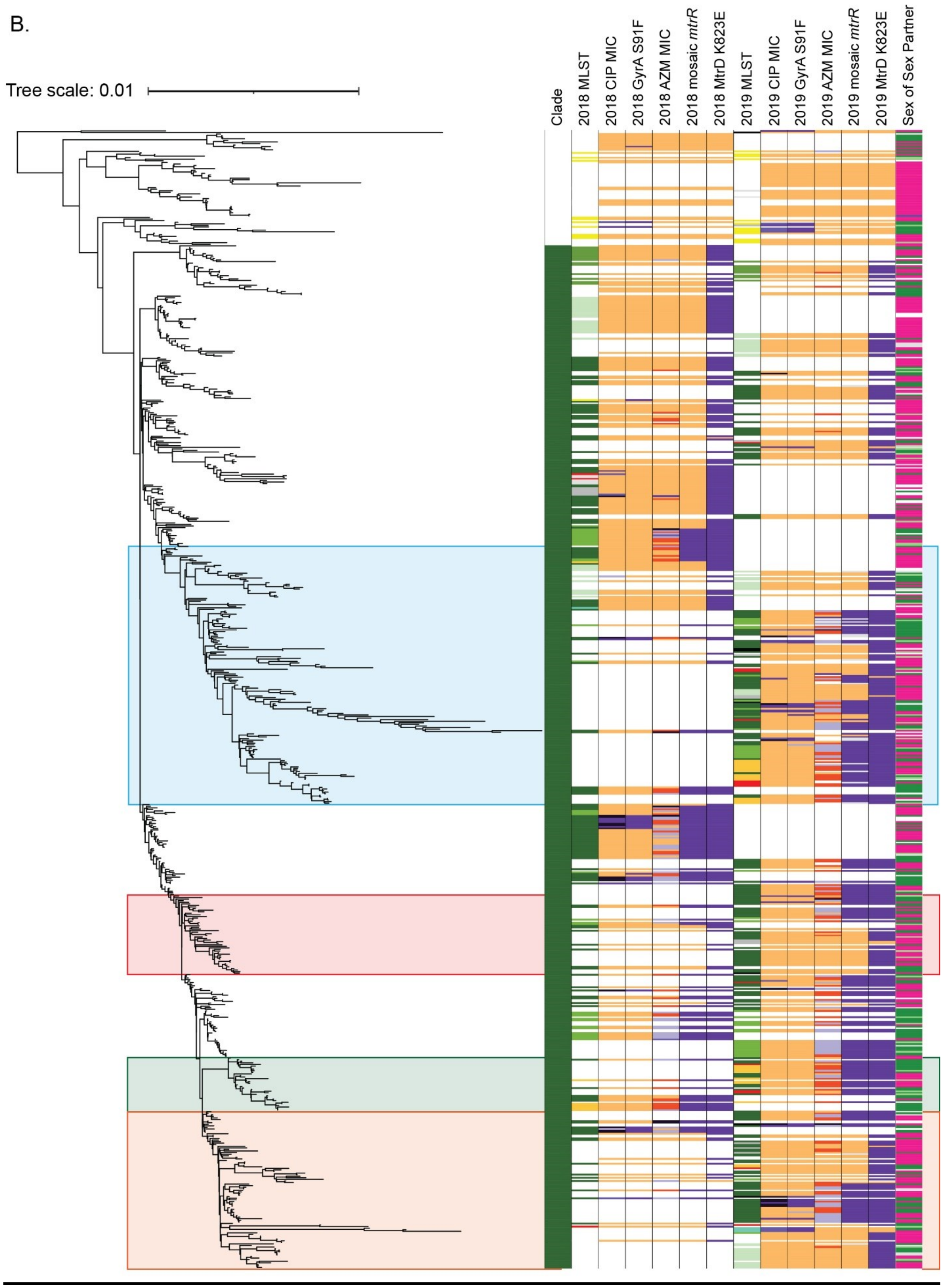
Tree scale: 0.01
Clade
2018 MLST
2018 CIP MIC
2018 GyrA S91F
2018 AZM MIC
2018 mosaic mtrR
2018 MtrD K823E
2019 MLST
2019 CIP MIC
2019 GyrA S91F
2019 AZM MIC
2019 mosaic mtrR
2019 MtrD K823E
Sex of Sex Partner

C.

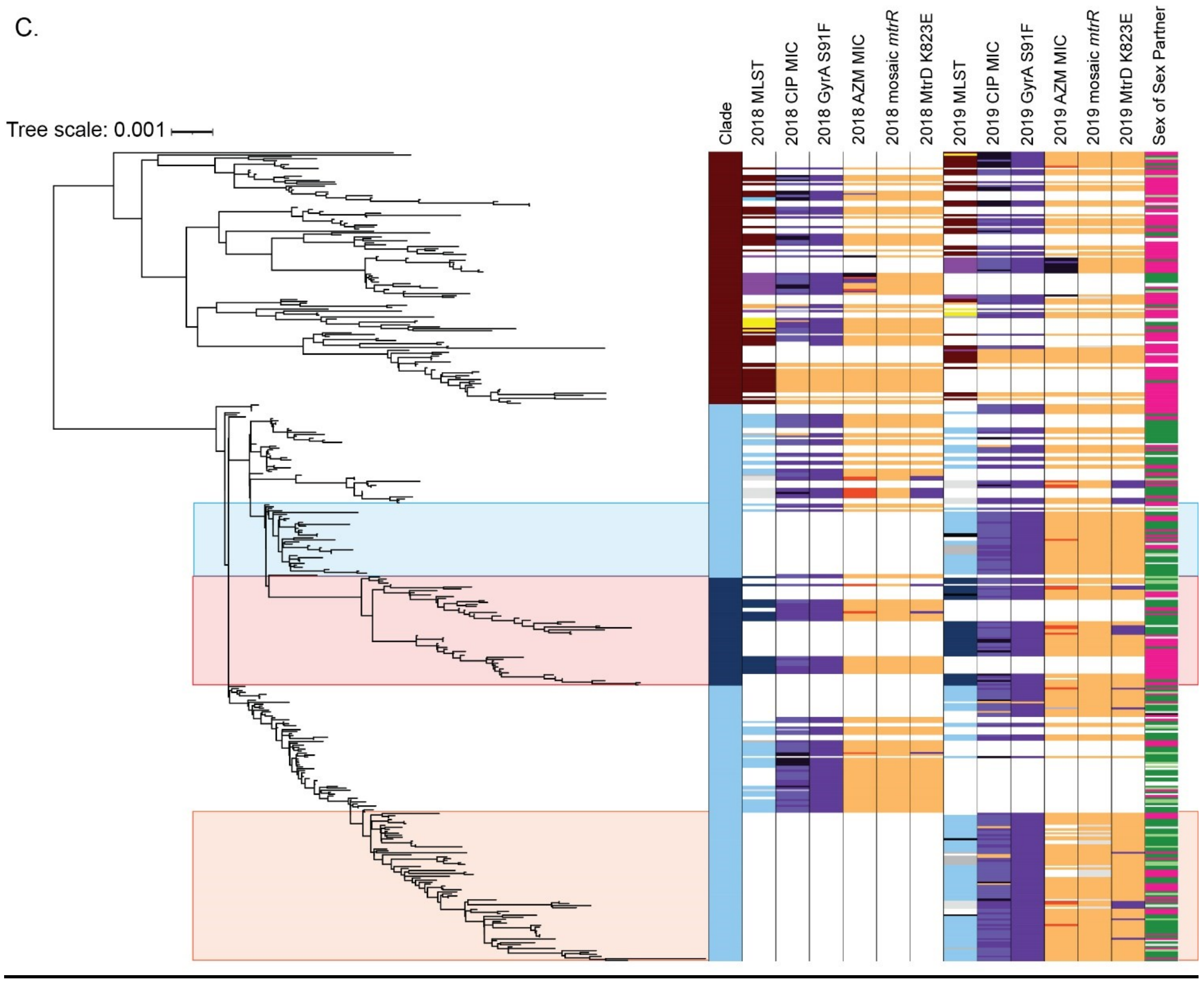
Tree scale: 0.001
Clade
2018 MLST
2018 CIP MIC
2018 GyrA S91F
2018 AZM MIC
2018 mosaic *mtrR*
2018 MtrD K823E
2019 MLST
2019 CIP MIC
2019 GyrA S91F
2019 AZM MIC
2019 mosaic *mtrR*
2019 MtrD K823E
Sex of Sex Partner

D.

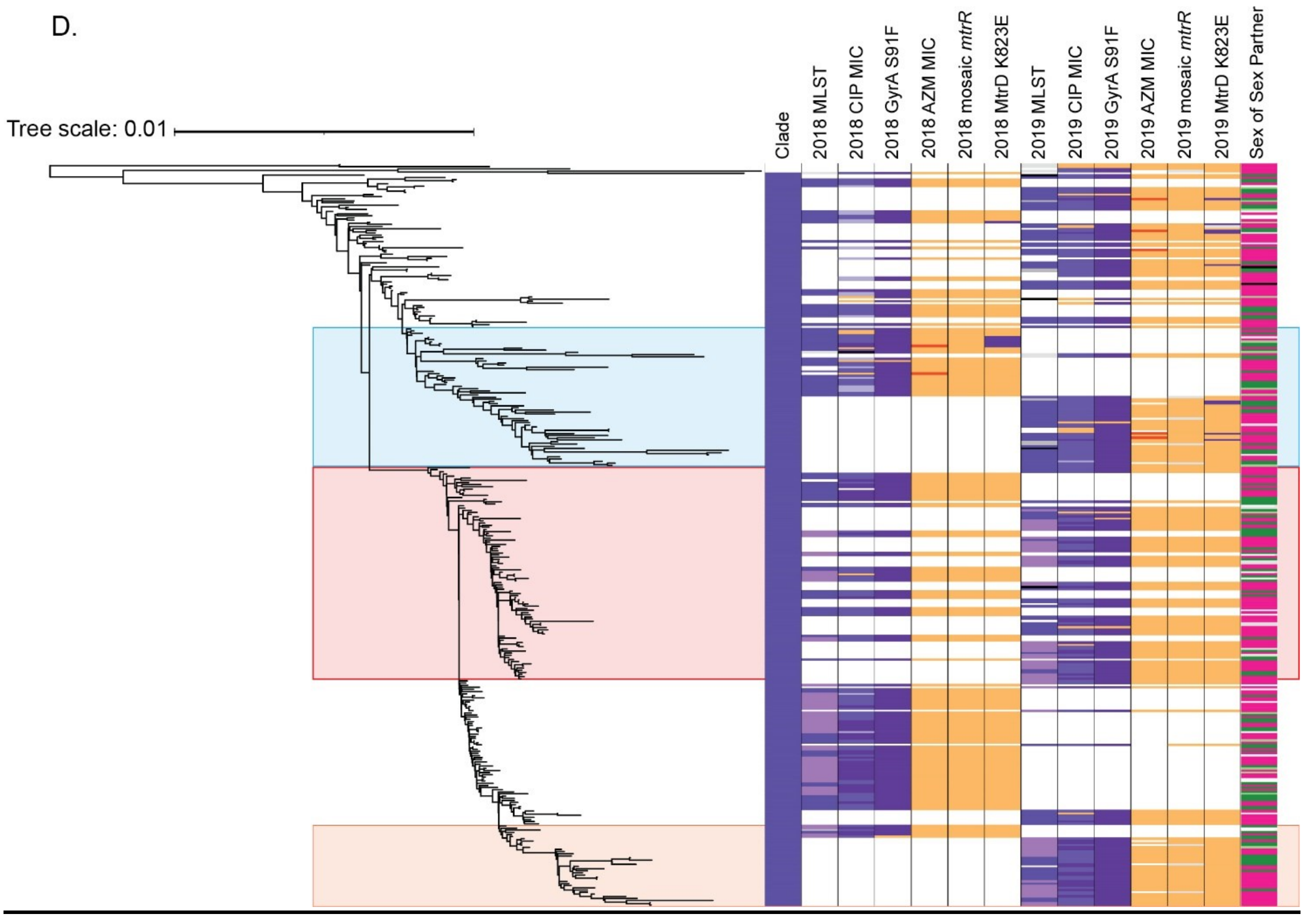

Supplemental Figure 2E. Maximum likelihood core-genome SNP phylogenetic reconstruction of 1710 GISP first 5 isolates per month per sentinel site from the United States in 2019.
Maximum likelihood core-genome SNP analyses defined the 1710 isolates into 22 clades ("Clade Label" left column) and color-defined in the left-most column. (Sub-clades for Clades 17 – 20 are labeled (17.1, 17.2, etc.) MLST STs are shown (with a color key on following page, listed in approximate order of appearance) and MLST STs ("MLST Label" in right-hand column). MLSTs with low representation are uncolored (white). Isolate susceptibility profiles are shown for AZM, CIP, PEN, TET, CFM, and CRO and colored according to MIC (susceptible (gold), elevated MIC (shades of purple)). The variants are represented as wild-type (gold) or mutant (orange, or light to dark purple). Sex of sex partner is represented as pink for men who have sex with women, dark green for men who have sex with men, light green for men who have sex with men and women, grey for unknown.

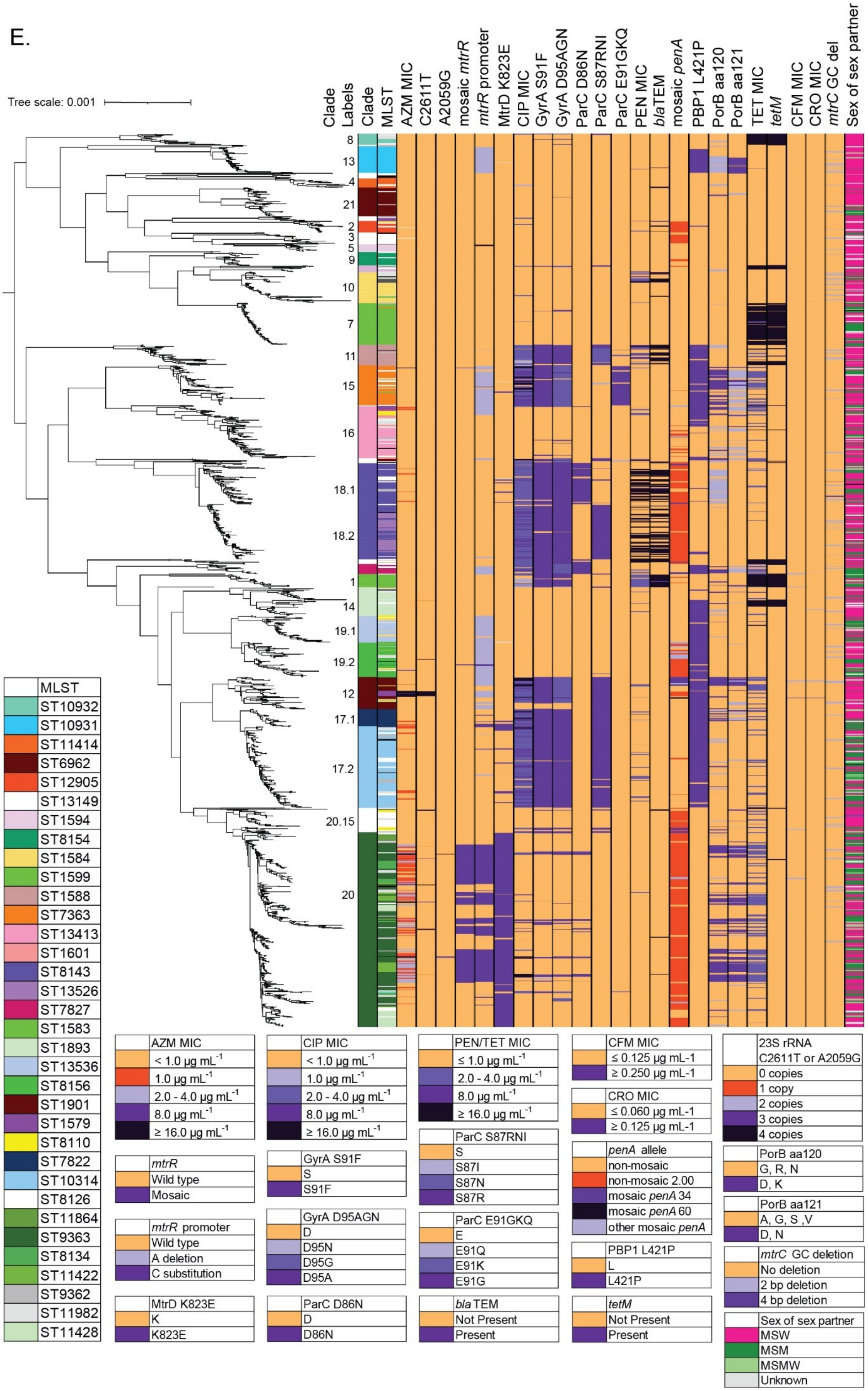
E.
Tree scale: 0.001
Clade Labels
Clade
MLST
AZM MIC
C2611T
A2059G
mosaic mtrR
mtrR promoter
MtrD K823E
CIP MIC
GyrA S91F
GyrA D95AGN
ParC D86N
ParC S87RNI
ParC E91GKQ
PEN MIC
blaTEM
mosaic penA
PBP1 L421P
PorB aa120
PorB aa121
TET MIC
tetM
CFM MIC
CRO MIC
mtrC GC del
Sex of sex partner
8
13
4
21
2
3
5
9
10
7
11
15
16
18.1
18.2
1
14
19.1
19.2
12
17.1
17.2
20.15
20
MLST
ST10932
ST10931
ST11414
ST6962
ST12905
ST13149
ST1594
ST8154
ST1584
ST1599
ST1588
ST7363
ST13413
ST1601
ST8143
ST13526
ST7827
ST1583
ST1893
ST13536
ST8156
ST1901
ST1579
ST8110
ST7822
ST10314
ST8126
ST11864
ST9363
ST8134
ST11422
ST9362
ST11982
ST11428
AZM MIC
< 1.0 μg mL-1
1.0 μg mL-1
2.0 - 4.0 μg mL-1
8.0 μg mL-1
≥ 16.0 μg mL-1
mtrR
Wild type
Mosaic
mtrR promoter
Wild type
A deletion
C substitution
MtrD K823E
K
K823E
CIP MIC
< 1.0 μg mL-1
1.0 μg mL-1
2.0 - 4.0 μg mL-1
8.0 μg mL-1
≥ 16.0 μg mL-1
GyrA S91F
S
S91F
GyrA D95AGN
D
D95N
D95G
D95A
ParC D86N
D
D86N
PEN/TET MIC
≤ 1.0 μg mL-1
2.0 - 4.0 μg mL-1
8.0 μg mL-1
≥ 16.0 μg mL-1
ParC S87RNI
S
S87I
S87N
S87R
ParC E91GKQ
E
E91Q
E91K
E91G
bla TEM
Not Present
Present
CFM MIC
≤ 0.125 μg mL-1
≥ 0.250 μg mL-1
CRO MIC
≤ 0.060 μg mL-1
≥ 0.125 μg mL-1
penA allele
non-mosaic
non-mosaic 2.00
mosaic penA 34
mosaic penA 60
other mosaic penA
PBP1 L421P
L
L421P
tetM
Not Present
Present
23S rRNA
C2611T or A2059G
0 copies
1 copy
2 copies
3 copies
4 copies
PorB aa120
G, R, N
D, K
PorB aa121
A, G, S ,V
D, N
mtrC GC deletion
No deletion
2 bp deletion
4 bp deletion
Sex of sex partner
MSW
MSM
MSMW
Unknown

Supplemental Table 3. Distribution of specimens by MLST and comparison between 2018 – 2019. Hypothesis testing for difference of isolate MLST between years were analyzed using Fisher's Exact Test with mid-P values from the Exact2x2 R package. All hypothesis testing used a significance level of α = 0.05. MLSTs with a representation in both years with a total of ≥5 isolates across the years are listed in the table.

MLST	2018(n)	2018(%)	2019(n)	2019 (%)	P-Value
ST9363	158	10.68%	200	11.70%	0.36704
ST10314	49	3.31%	113	6.61%	***0.00002***
ST8143	94	6.36%	106	6.20%	0.85473
ST1599	56	3.79%	77	4.50%	0.31466
ST7363	58	3.92%	57	3.33%	0.37625
ST13526	53	3.58%	55	3.22%	0.56865
ST6962	37	2.50%	50	2.92%	0.46892
ST10931	32	2.16%	50	2.92%	0.17781
ST1901	48	3.25%	48	2.81%	0.47181
ST1584	59	3.99%	45	2.63%	***0.03239***
ST8156	43	2.91%	43	2.51%	0.49668
ST1893	39	2.64%	43	2.51%	0.82708
ST11422	35	2.37%	41	2.40%	0.95652
ST11428	32	2.16%	41	2.40%	0.66353
ST13413	24	1.62%	41	2.40%	0.12384
ST1588	41	2.77%	37	2.16%	0.27063
ST13536	30	2.03%	36	2.11%	0.88240
ST7822	20	1.35%	33	1.93%	0.20653
ST1583	19	1.28%	33	1.93%	0.15380
ST8126	7	0.47%	31	1.81%	***0.00036***
ST8134	7	0.47%	27	1.58%	***0.00203***
ST8154	29	1.96%	23	1.35%	0.17543
ST1579	23	1.56%	18	1.05%	0.21495
ST12905	22	1.49%	15	0.88%	0.11349
ST11982	3	0.20%	14	0.82%	***0.01665***
ST1601	26	1.76%	13	0.76%	***0.01135***
ST7827	21	1.42%	13	0.76%	0.07462
ST1594	16	1.08%	13	0.76%	0.34830
ST1580	11	0.74%	13	0.76%	0.96189
ST11414	22	1.49%	12	0.70%	***0.03345***
ST14253	11	0.74%	12	0.70%	0.88790
ST9902	3	0.20%	12	0.70%	***0.04134***
ST10932	15	1.01%	11	0.64%	0.25462
ST11706	7	0.47%	11	0.64%	0.53754
ST13532	6	0.41%	11	0.64%	0.37326
ST11864	15	1.01%	10	0.58%	0.17901
ST9362	5	0.34%	10	0.58%	0.32606
ST1587	6	0.41%	9	0.53%	0.63553
ST12093	6	0.41%	9	0.53%	0.63553

ST11648	3	0.20%	9	0.53%	0.14839
ST1918	9	0.61%	8	0.47%	0.59567
ST10317	9	0.61%	8	0.47%	0.59567
ST13527	6	0.41%	7	0.41%	0.99249
ST13149	2	0.14%	7	0.41%	0.16256
ST13736	1	0.07%	7	0.41%	0.06069
ST11986	4	0.27%	6	0.35%	0.70592
ST12467	3	0.20%	6	0.35%	0.46039
ST11418	2	0.14%	6	0.35%	0.25089
ST13533	1	0.07%	6	0.35%	0.10235
ST1582	1	0.07%	6	0.35%	0.10235
ST8149	12	0.81%	5	0.29%	***0.04999***
ST11413	9	0.61%	5	0.29%	0.19327
ST11184	7	0.47%	5	0.29%	0.42420
ST7359	6	0.41%	5	0.29%	0.60125
ST13732	2	0.14%	5	0.29%	0.37961
ST11423	8	0.54%	4	0.23%	0.17461
ST8110	6	0.41%	4	0.23%	0.40985
ST12546	4	0.27%	4	0.23%	0.84223
ST12462	14	0.95%	3	0.18%	***0.00302***
ST11967	9	0.61%	3	0.18%	0.05368
ST12548	4	0.27%	3	0.18%	0.59237
ST13292	2	0.14%	3	0.18%	0.80357
ST13733	2	0.14%	3	0.18%	0.80357
ST11713	2	0.14%	3	0.18%	0.80357
ST11516	8	0.54%	2	0.12%	***0.03899***
ST8163	6	0.41%	2	0.12%	0.12358
ST1903	5	0.34%	2	0.12%	0.21306
ST11181	10	0.68%	1	0.06%	***0.00310***
ST7371	7	0.47%	1	0.06%	***0.02393***
ST1585	7	0.47%	1	0.06%	***0.02393***
ST13489	6	0.41%	1	0.06%	***0.04638***
ST1600	5	0.34%	1	0.06%	0.08869

Supplemental Table 4A. Distribution of specimens by MIC to AZM and comparison between 2018 – 2019. Antimicrobial resistance MIC categorization between years used Pearson's Chi-Squared Test using base R and without continuity correction. All hypothesis testing used a significance level of $\alpha = 0.05$.

From 2018 – 2019, the percent of isolates with AZM MIC 2.0 µg mL^{-1} increased by 1.4% (p-value 0.0388); more significantly the percent of isolates with AZM MIC 1.0 µg mL^{-1} increased by 3.2% (p-value 0.0001); the percent of isolates with medium-high level AZM MIC (≥4.0 µg mL^{-1}) showed no change.

AZM_MIC (µg mL^{-1})	WGS_2018 Count	WGS_2018 Percent	WGS_2019 Count	WGS_2019 Percent	P-Value
<=0.5	1350	91.3%	1480	86.5%	**2.52E-05**
1	63	4.3%	129	7.5%	**1.01E-04**
2	45	3.0%	76	4.4%	0.038802
>=4	21	1.4%	25	1.5%	0.920774

Supplemental Table 4B. Distribution of specimens by MIC to PEN and comparison between 2018 – 2019 The percent of isolates with elevated MIC 2.0 µg mL^{-1} or MIC ≥4.0 µg mL^{-1} to PEN showed no significant change between 2018 and 2019.

PEN_MIC (µg mL^{-1})	WGS_2018 Count	WGS_2018 Percent	WGS_2019 Count	WGS_2019 Percent	P-Value
<=1	1289	87.2%	1488	87.0%	0.909132
2	77	5.2%	82	4.8%	0.594970
>=4	113	7.6%	140	8.2%	0.568808

Supplemental Table 4C. Distribution of specimens by MIC to TET and comparison between 2018 – 2019 The percent of isolates with elevated MIC 2.0-4.0 µg mL^{-1} or MIC ≥8.0 µg mL^{-1} to TET showed no significant change between 2018 and 2019.

TET_MIC (µg mL^{-1})	WGS_2018 Count	WGS_2018 Percent	WGS_2019 Count	WGS_2019 Percent	P-Value
<=1	1099	74.3%	1238	72.4%	0.224303
2 - 4	228	15.4%	305	17.8%	0.067708
>=8	152	10.3%	167	9.8%	0.631412

Supplemental Table 4D. Distribution of specimens by MIC to CFM and comparison between 2018 – 2019 The percent of isolates with elevated MIC 0.250 µg mL^{-1} showed no significant change between 2018 and 2019.

CFM_MIC (µg mL^{-1})	WGS_2018 Count	WGS_2018 Percent	WGS_2019 Count	WGS_2019 Percent	P-Value
<=0.125	1474	99.66%	1702	99.53%	0.566272
>=0.250	5	0.34%	8	0.47%	0.566272

Supplemental Table 4E. Distribution of specimens by MIC to CRO and comparison between 2018 – 2019
The percent of isolates with elevated MIC 0.125 µg mL^{-1} showed no significant change between 2018 and 2019.

CRO_MIC (µg mL^{-1})	WGS_2018 Count	WGS_2018 Percent	WGS_2019 Count	WGS_2019 Percent	P-Value
<=0.06	1475	99.7%	1701	99.47%	0.907261
>=0.125	4	0.27%	5	0.29%	0.907261

Statistics

Supplemental Table 5. Distribution of isolates per clade based on sex of sex partner. Analysis of sex of sex partner categorization into clades and subclades were tested using Fisher's Exact Test with mid-P values. All hypothesis testing used a significance level of α = 0.05.
Subclades 17.1, 17.2, 18.2, 18.2, 19.1, 19.2 are a subdivision of the original clade based on internal branching. See Supplemental Figure 2. Clade 20 was subdivided into Subclade 20.1 and 20.2 based on absence or presence of mosaic *mtr* operon.
Subclade 20.2 represented a patient population of 42% (82/192) MSW and 42% (81/192) MSM (which represents statistically higher percent MSM than the overall population, p-value 4.789 e-06).

Clade	MSW	%	p-value		Clade	MSM	%	p-value		Clade	MSMW	%	p-value
0	68	80%	**2.1197E-05**		0	10	12%	**4.0695E-04**		0	4	4.7%	5.6632E-01
1	3	13%	**3.8496E-06**		1	15	63%	**3.7232E-04**		1	3	12.5%	2.5003E-01
2	14	64%	6.4446E-01		2	3	14%	1.4272E-01		2	2	9.1%	5.6757E-01
3	12	57%	8.8557E-01		3	3	14%	1.7588E-01		3	0	0.0%	2.5198E-01
4	17	94%	**9.2955E-04**		4	0	0%	**3.0141E-03**		4	0	0.0%	3.0715E-01
5	12	92%	**1.0500E-02**		5	0	0%	**1.5225E-02**		5	0	0.0%	4.2689E-01
7	33	41%	**1.4820E-03**		7	31	39%	**2.4611E-02**		7	7	8.8%	3.6406E-01
8	21	100%	**1.2224E-05**		8	0	0%	**1.1375E-03**		8	0	0.0%	2.5198E-01
9	21	84%	**7.6429E-03**		9	0	0%	**3.0922E-04**		9	0	0.0%	1.9341E-01
10	37	62%	6.3026E-01		10	11	18%	1.0343E-01		10	3	5.0%	7.2366E-01
11	29	73%	7.0101E-02		11	7	18%	1.5204E-01		11	2	5.0%	7.9963E-01
12	53	87%	**1.3132E-06**		12	4	7%	**3.7847E-05**		12	2	3.3%	3.3734E-01
13	44	86%	**1.7655E-05**		13	2	4%	**1.4229E-05**		13	2	3.9%	5.1863E-01
14	37	69%	1.3318E-01		14	8	15%	**2.9087E-02**		14	0	0.0%	**2.7858E-02**
15	53	70%	**4.2498E-02**		15	13	17%	**3.4221E-02**		15	2	2.6%	1.6808E-01
16	62	62%	4.8058E-01		16	24	24%	4.3515E-01		16	11	11.0%	6.6197E-02
17	63	33%	**1.2834E-13**		17	99	52%	**1.1942E-14**		17	14	7.4%	5.0519E-01
18	119	64%	9.3499E-02		18	46	25%	4.1186E-01		18	6	3.2%	5.7257E-02
19	55	47%	**6.7769E-03**		19	45	38%	**8.8315E-03**		19	10	8.5%	3.2438E-01
20	188	51%	**3.9268E-04**		20	123	33%	**6.5794E-03**		20	35	9.4%	**8.0320E-03**
21	42	76%	**5.6104E-03**		21	11	20%	2.0972E-01		21	2	3.6%	4.3829E-01
22	19	48%	1.5637E-01		22	14	35%	2.8585E-01		22	3	7.5%	7.1192E-01

Sub-clade	MS W	%	p-value		Sub-clade	MS M	%	p-value		Sub-clade	MSM W	%	p-value
17.1	17	50%	3.1138E-01		17.1	11	32%	5.1334E-01		17.1	3	8.8%	5.2767E-01
17.2	46	30%	**2.8186E-14**		17.2	88	57%	**7.0203E-16**		17.2	11	7.1%	6.5587E-01
18.1	46	58%	8.3516E-01		18.1	24	30%	5.9185E-01		18.1	4	5.0%	6.6295E-01
18.2	73	70%	**1.7999E-02**		18.2	22	21%	1.2160E-01		18.2	2	1.9%	**3.8271E-02**
19.1	23	45%	5.0730E-02		19.1	19	37%	1.2080E-01		19.1	4	7.8%	6.2036E-01
19.2	32	48%	6.9937E-02		19.2	26	39%	**3.9600E-02**		19.2	6	9.0%	3.7009E-01
20.1	106	59%	9.1378E-01		20.1	42	24%	2.2586E-01		20.1	16	9.0%	1.3613E-01
20.2	81	42%	**2.6389E-06**		20.2	81	42%	**4.7894E-06**		20.2	19	9.8%	**4.5606E-02**
21	42	76%	**5.6104E-03**		21	11	20%	2.0972E-01		21	2	3.6%	4.3829E-01

Supplemental Table 6. Chi Square for Antibiotics with elevated MICs and the associated genomic variants
Of those isolates in clade 20 which carried mosaic *mtr* operon (defined as subclade 20.2), 84.9% (163 / 192) of isolates had an AZM MIC 1.0-2.0 µg mL^{-1} (chi-square for AZM MIC 1.0-2.0 µg mL^{-1} mosaic *mtr*, $\chi 2(1, N=1685) = 1121.574$, $p < 0.01$)

AZM
MIC 1.0 - 2.0 µg mL^{-1}

	MIC (1.0 – 2.0 µg mL^{-1})	Susceptible	Marginal row totals
mosaic *mtrR* present	**163**	**22**	185
mosaic *mtrR* absent	**42**	**1458**	1500
Marginal column totals	205	1480	1685

PPV	88.12
NPV	97.20
Sensitivity	79.51
Specificity	98.51

Out of 1710 isolates in the dataset, 25 isolates (1.5%) had high-level AZM^{em} (MIC >= 4.0 µg mL^{-1}) which associated with 23S rRNA variants (chi-square for AZM^{em} MIC >=4.0 µg mL^{-1}, 23S rRNA, $\chi 2(1, N=1710) = 1109.0647$, $p < .01$).

Medium to high-level AZM^{em}
(MIC >= 4.0 µg mL^{-1})

	Elevated MIC (MIC >=4.0 µg mL^{-1})	MIC <4.0 µg mL^{-1}	Marginal row totals
23S rRNA (C2611T or A2059G)	**21**	**6**	27
23S rRNA wild type	**4**	**1679**	1683
Marginal column totals	25	1685	1710

PPV	77.78
NPV	99.76
Sensitivity	84.00
Specificity	99.64

CIP resistance (MIC >= 1.0 µg mL^{-1}) was predominantly determined by GyrA S91F variant (chi-square for CIP MIC ≥ 1 µg mL^{-1}, GyrA, $\chi 2(1, N=1710) = 1510.9567$, $p < .01$).

	Resistance (MIC >=1.0 µg mL^{-1})	Susceptible	Marginal row totals
Mutant GyrA S91F	**601**	**37**	638
Wild type GyrA S91	**11**	**1061**	1072
Marginal column totals	612	1098	1710

PPV	94.20
NPV	98.97
Sensitivity	98.20
Specificity	96.63

PEN resistance (MIC >= 2.0 µg mL^{-1}) was observed in isolates carrying the *blaTEM* plasmid (chi-square for PEN MIC MIC ≥ 2 µg mL^{-1}, *blaTEM*, $\chi 2(1, N=1710) = 1060.7114, p < .01$).

blaTEM	Resistance (MIC >=2.0 µg mL^{-1})	Susceptible	Marginal row totals
bla_{TEM} present	**158**	**13**	171
bla_{TEM} absent	**64**	**1475**	1539
Marginal column totals	222	1488	1710

PPV	92.40
NPV	95.84
Sensitivity	71.17
Specificity	99.13

TET resistance with high MIC (~8 - 64 µg mL^{-1}) carried the *tetM* plasmid (chi-square for TET^R MIC >=8.0 µg mL^{-1}, *tetM*, $\chi 2(1, N=1710) = 1676.0545, p < .01$).

	Resistance MIC (MIC >=8.0 µg mL^{-1})	MIC <8.0 µg mL^{-1}	Marginal row totals
tetM present	**165**	**1**	166
tetM absent	**2**	**1542**	1544
Marginal column totals	167	1543	1710

PPV	99.40
NPV	99.87
Sensitivity	98.80
Specificity	99.94

Supplemental Table 7A. Characteristics of mosaic *mtr* operon subclades.

AZM MIC ($\mu g\ mL^{-1}$)	**Subclade 5 (n=165)**	**Subclade 2 (n=47)**	**Subclade 6 (n=21)**	**Subclade 1 (n=124)**
<1	94.5% (156/165)	14.9% (7/47)	14.3% (3/21)	8.9% (11/124)
1	4.8% (8/165)	68.1% (32/47)	28.6% (6/21)	39.5% (49/124)
2	0% (0/165)	17.0% (8/47)	42.9% (9/21)	46.8% (58/124)
>2	0.6% (1/165)	0% (0/47)	14.3% (3/21)	4.8% (6/124)
Top MLST	9363, 11428	8134, 9363	9363	9363, 11422
% ID compared to FA19	94.59%	93.55%	92.93%	92.48%
**Top BLASTn hits (% ID)**				
mtrD only	*mtrD* variant C (100%)<br>*N. gonorrhoeae* FQ02 (100%)<br>*N. gonorrhoeae* FQ04 (99.97%)<br>*mtrD* variant A (99.25%)<br>*N. cinerea* NCTC10294 and FDAARGOS_871 (96.41%)<br>*mtrD* variant B (94.60%)	*mtrD* variant B (99.34%)<br>*mtrD* variant A (94.23%)<br>*N. gonorrhoeae* FQ02 (94.14%)<br>*mtrD* variant C (94.13%)<br>*N. cinerea* NCTC10294 and FDAARGOS_871 (94.13%)<br>*N. gonorrhoeae* FQ04 (94.11%)	*mtrD* variant B (100%)<br>*mtrD* variant A (95%)<br>*N. cinerea* NCTC10294 and FDAARGOS_871 (94.63%)<br>*mtrD* variant C (94.60%)<br>*N. gonorrhoeae* FQ02 (94.60%)<br>*N. gonorrhoeae* FQ04 (94.57%)	*mtrD* variant B (100%)<br>*mtrD* variant A (95%)<br>*N. cinerea* NCTC10294 and FDAARGOS_871 (94.63%)<br>*mtrD* variant C (94.60%)<br>*N. gonorrhoeae* FQ02 (94.60%)<br>*N. gonorrhoeae* FQ04 (94.57%)
mtrR-mtrCDE Whole operon	*N. gonorrhoeae* FQ02 (100%)<br>*N. gonorrhoeae* FQ04 (99.94%)<br>*N. gonorrhoeae* 34769 (94.75%)	*N. gonorrhoeae* FQ02 (94.88%)<br>*N. gonorrhoeae* FQ04 (94.88%)<br>*N. polysaccharea* M18661 (94.75%)<br>*N. lactamica* 020-06 (94.03%)<br>*N. lactamica* NCTC10617 (93.94%)<br>*N. lactamica* Y92-1009 (93.81%)<br>*N. meningitidis* M01-240355 (93.67%)	*N. gonorrhoeae* FQ02 (95.02%)<br>*N. gonorrhoeae* FQ04 (94.99%)<br>*N. polysaccharea* M18661 (94.52%)<br>*N. meningitidis* M01-240355 (94.14%)<br>*N. lactamica* NCTC10617 (94.07%)<br>*N. lactamica* M17106 (93.99%)<br>*N. lactamica* Y92-1009 (93.96%)<br>*N. lactamica* 020-06 (93.92%)	*N. polysaccharea* M18661 (94.76%)<br>*N. gonorrhoeae* FQ02 (94.58%)<br>*N. gonorrhoeae* FQ04 (94.58%)<br>*N. meningitidis* M01-240355 (94.25%)<br>*N. lactamica* NCTC10617 (94.19%)<br>*N. lactamica* Y92-1009 (94.10%)<br>*N. lactamica* M17106 (94.09%)<br>*N. lactamica* 020-06 (94.03%)<br>*N. cinerea* NCTC10294 (92.90%)

Supplemental Table 7B. Seven sequences had NF calls for *mtrD* from AMR Profiler. All have premature stop codons in *mtrD* occurring at different places in the sequence. The coverage for GCWGS-5545 is quite low, thus the variant causing the premature stop could potentially be a sequencing/assembly error.
The seven isolates were varied in MLST type and were distributed around the phylogenetic tree. All seven have AZM MIC 0.015 – 0.030 µg mL^{-1}.

	Coverage				
	mtrR	*mtrD*	*MLST*	*Clade*	*AZM MIC* µg mL^{-1}
GCWGS-11626	31.4	31.3	7827	22.3	0.03
GCWGS-6735	56.8	57.9	10931	13	0.015
GCWGS-10946	40.2	40.2	16152	10	0.015
GCWGS-7665	44.2	26.3	8143	18.1	0.03
GCWGS-5545	8.97	15	11648	15	0.03
GCWGS-9099	41.5	37.1	8156	19.2	0.03
GCWGS-9146	48.4	44.6	13413	16	0.03

Supplemental Table 8. Ten isolates in clade 20 (seven of them ST9363) had a range of one to four copies of the C2611T variant, and the AZM MICs ranged from 0.125 to ≥16 µg mL^{-1}. The remainder of the isolates with C2611T variants had AZM MICs from 0.5 to 8 µg mL^{-1} and were in MLST ST1580, ST1893, ST10932 and ST8156 (2 isolates).

GCWGS_ID	AZM MIC (µg mL^{-1})	23S rRNA variant	23S frequency	Count C2611T	MLST
GCWGS-8385	16	A2059G	0.627	2	14101
GCWGS-12081	16	A2059G	1	4	9363
GCWGS-8386	16	C2611T	1	4	1579
GCWGS-8828	16	C2611T	1	4	1579
GCWGS-8829	16	C2611T	1	4	1579
GCWGS-9898	16	C2611T	1	4	1579
GCWGS-11557	16	C2611T	1	4	1579
GCWGS-8337	16	C2611T	1	4	1579
GCWGS-6240	16	C2611T	1	4	1579
GCWGS-8437	8	C2611T	1	4	1579
GCWGS-9827	16	C2611T	1	4	1579
GCWGS-14263	8	C2611T	1	4	1579
GCWGS-7519	0.5	C2611T	0.054	1	8134
GCWGS-6397	2	C2611T	0.257	1	9363
GCWGS-8916	2	C2611T	0.219	1	14101
GCWGS-9270	0.5	C2611T	0.611	2	9362
GCWGS-11978	0.125	C2611T	0.542	2	9363
GCWGS-9459	4	C2611T	0.494	2	9363
GCWGS-11166	16	C2611T	0.777	3	9363
GCWGS-12600	8	C2611T	0.681	3	9363
GCWGS-6717	16	C2611T	1	4	9363
GCWGS-11509	8	C2611T	1	4	9363
GCWGS-6463	0.5	C2611T	0.190	1	1893
GCWGS-11528	4	C2611T	1	4	1580
GCWGS-11115	8	C2611T	1	4	8156
GCWGS-9705	4	C2611T	1	4	8156
GCWGS-8244	4	C2611T	1	4	10932

Supplemental Figure 3. Count of isolates per MLST which carried 23S rRNA variants. Histograms are colored based on copy number.
Clade 20 includes MLST 9363, 8134, 9362, 14101, 11428, 11422, 11982.

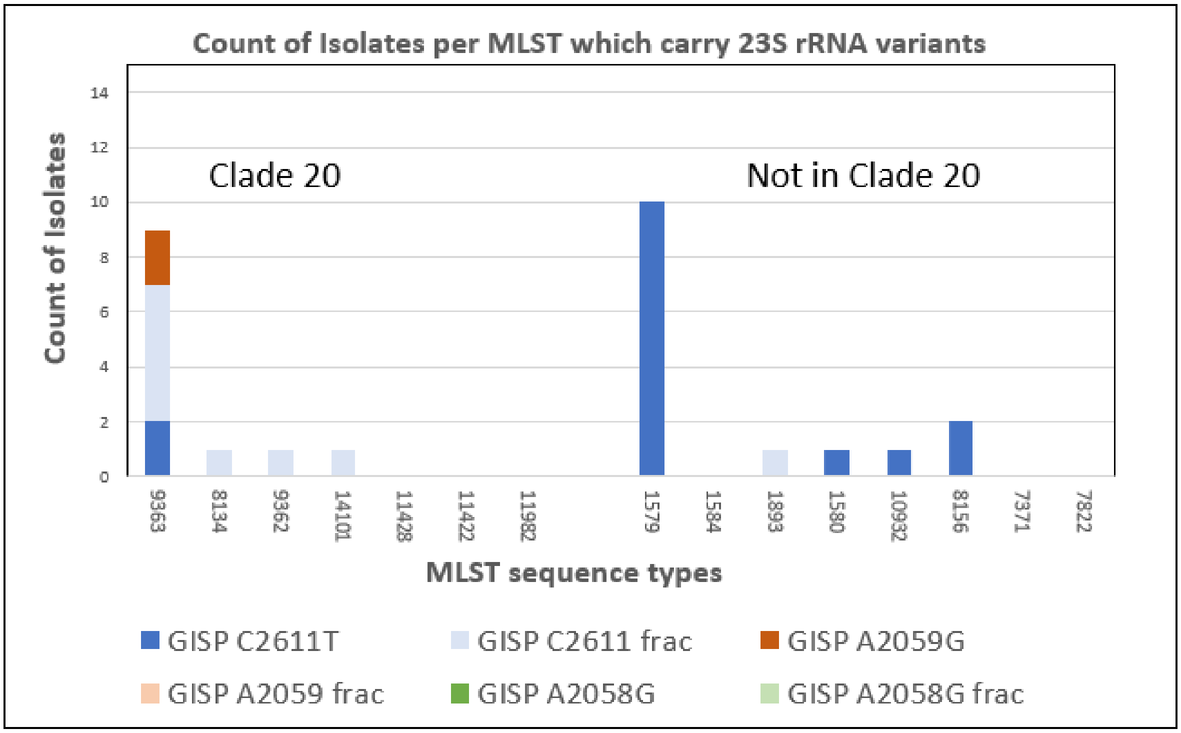

Supplemental Table 9. Isolates with CFM^{em} or CRO^{em} were phylogenetically diverse and carried various *penA* alleles.

GCWGS_ID	CFM MIC (µg mL^{-1})	CRO MIC (µg mL^{-1})	MLST	*penA* #	*penA* ngSTAR *	Subclade
GCWGS-10753	1	1	1901	60.001	60.001	12
GCWGS-9190	0.25	0.125	1901	34.001	34.001	12
GCWGS-6545	0.25	0.06	1901	34.001	34.001	12
GCWGS-10730	0.25	0.03	7827	34.001	34.001	22.3
GCWGS-11749	0.125	0.125	7827	13.001	13.001	22.3
GCWGS-9277	0.125	0.125	1893	9.001	202.001	14
GCWGS-9281	0.125	0.125	1893	9.001	202.001	14
GCWGS-9756	0.25	0.06	7363	10.001	10.001	0
GCWGS-9973	0.25	0.06	1600	27.002	205.001	0
GCWGS-11110	0.25	0.03	1580	34.001	34.001	0
GCWGS-7410	0.25	0.03	9363	67.001	198.002	20.2

# penA allele call as determined by AMR-Profiler and Typing Tool 2.9.2-dev (08/2020 – 06/2021), based on pubMLST.org penA allele database 06/2020.
* penA allele call as determined by ngSTAR (pyngstar), database (https://ngstar.canada.ca, 11/29/2021).

Supplemental Table 9B. Isolates with CFM^{em} or CRO^{em} from 2014-2016, 2017 and 2018 U.S. GISP studies were phylogenetically diverse and carried various *penA* alleles. In each year, ST1901 (and phylogenetically related sequence types ST9365 and ST8110, (**bold** in the table)) included isolates with elevated MIC to CFM (as defined by the cut-offs in Table 1, MIC ≥0.25 µg mL^{-1}, CFM^{em}) and to CRO (as defined by the cut-offs in Table 1, MIC ≥0.125 µg mL^{-1}, CRO^{em}) (21, 18, 19)

	count CRO	Clade	MLST	count CFM	Clade	MLST	Ref
2014 – 2016<br>N=649	25	**B**<br>I	**1901 (9), 9365 (1)**<br>7827 (8)	46	**B**<br>C<br>G<br>other	**1901 (23), 9365 (4) 8110 (1)**<br>1579 (2)<br>7363 (4)<br>7827 (3)	21
2017<br>N=410	6	**C**<br>G	**1901 (2),**<br>7827 (3)	14	**C**<br>H	**1901 (7), 9365 (2), 8110 (1)**<br>8143 (1)	18
2018<br>N=1479	4	9<br>**14**<br>16	ST7827 (2)<br>**ST9365** (1)<br>ST9363 (1)	5	 **14**<br>19<br>other	7827<br>**ST8110 (1), ST9365 (1)**<br>ST1580 (1), ST7363 (1)<br>ST1893 (1)	19
2019<br>N=1710	5	**12**<br>22.3<br>14	**1901 (2)**<br>7827 (1)<br>1893 (2)	8	**12**<br>20.2<br>22.3<br>other	**1901 (3)**<br>9363 (1)<br>7827 (1)<br>1600 (1), 1580(1), 7363 (1)	This study

Specimen selection for sequencing of GISP isolates for 2014-2016 (n=649) and 2017 (n=410) were based on an elevated MICs to AZM, CFM and/or CRO, and did not represent a strict surveillance-based dataset. Thus, the prevalence of CRO^{em} and CFM^{em} could not be compared with 2018 and 2019 surveillance data sets.

Supplemental Table 10.

porB1a (12.8%, 219/1710)	**aa120**		**aa121**	
	G	49% (108/219)	G	100% (219/219)
	D	51% (111/219)		

porB1b 85.7% (1465/1710)	**aa120**		**aa121**	
G	78%	(1139/1465)	0.6%	(9/1465)
A			55%	(812/1465)
V			0.2%	(3/1465)
S			23%	(337/1465)
N	1.4%	(21/1465)	12%	(182/1465)
K	17%	(242/1465)		
D	4%	(63/1465)	8%	(122/1465)

Supplemental Figure 4A. Multilocus sequence type distribution of the 1710 GISP 1st 5 isolates per month per sentinel site from 2019 in the United States. The 37 most prevalent STs (that include >10 isolates) are shown. Proportions of susceptibilities to CIP are shown by colors indicated. Eight MLST types account for 72.7% (445/612) of all CIP^{R} isolates.

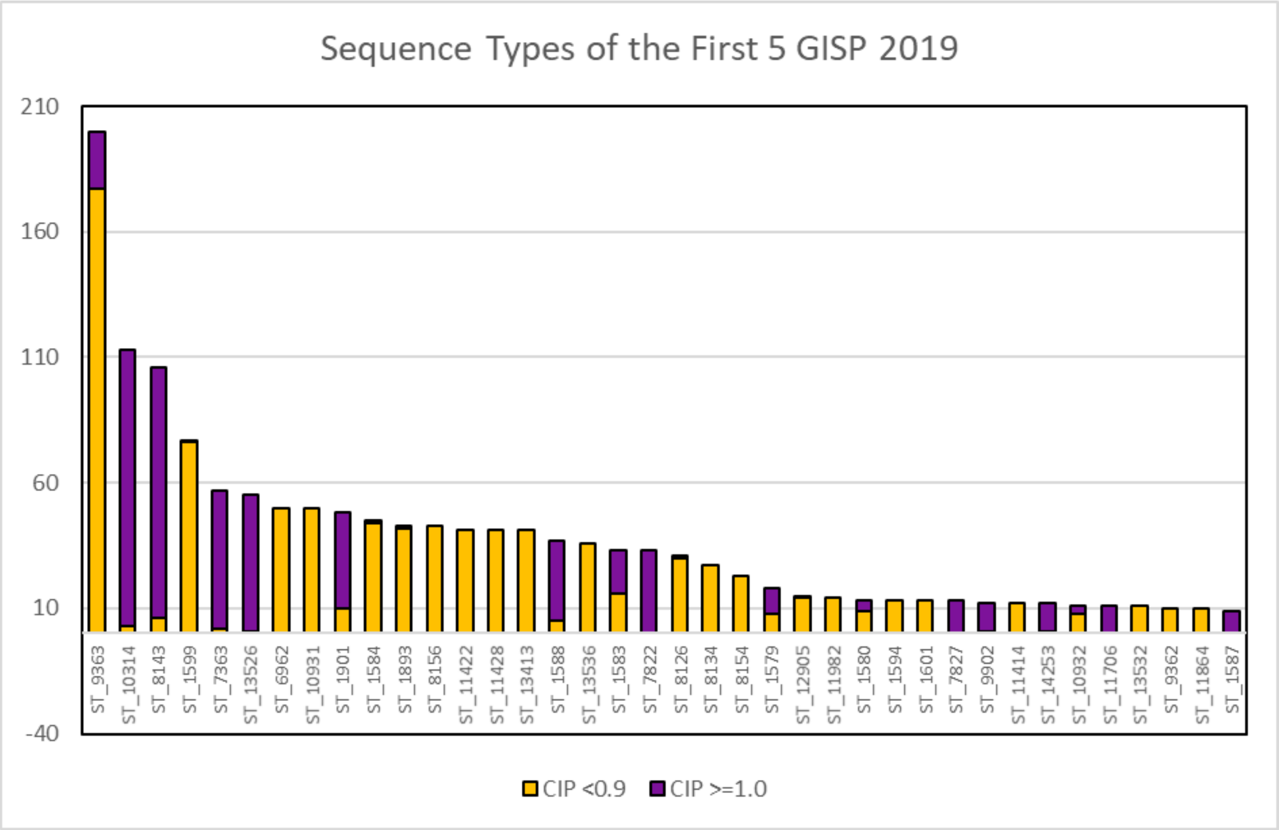

Supplemental Figure 4B. Proportions of susceptibilities to PEN are shown by colors indicated. One-half of the PEN^R isolates MIC >=2.0 μg mL^{-1} (50.4%, 112/222) grouped in clade 18 (ST8143 and ST13526), and clade 11 (ST1588) (Figure 4B); 48.6% (90/185) of clade 18 and 57.5% (23/40) of clade 11, ST1588, carry *blaTEM*, resulting in PEN MICs ranging from 2-16 μg mL^{-1}. 100% (23/23) of clade 1, ST1583, carry the *blaTEM* plasmid, but have a lower range of MICs at 1.0-4.0 μg mL^{-1}.

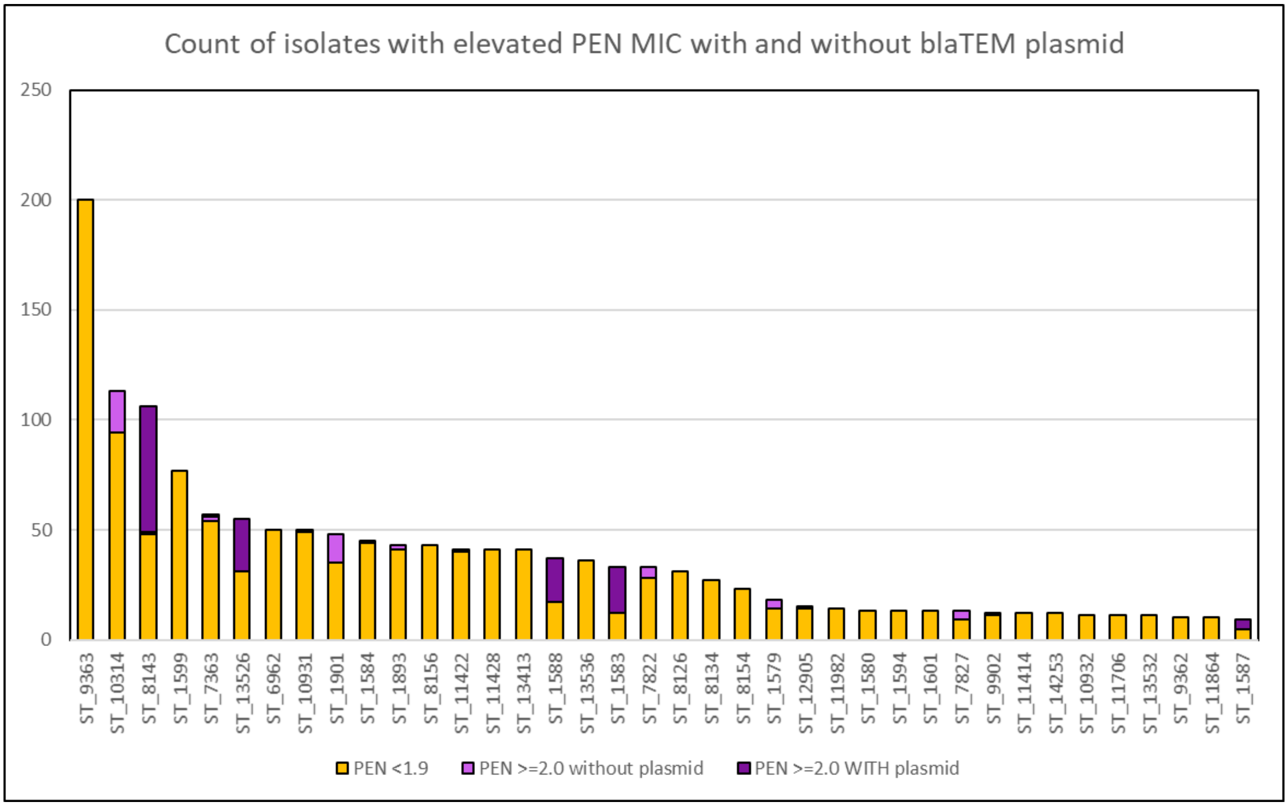

Supplemental Figure 4C. Proportions of susceptibilities to TET are shown by colors indicated. The TET^R isolates with MIC ~8-64 µg mL^{-1} carried the *tetM* plasmid and were predominately in clade 7 (ST1599 (69/80)), clade 1 (ST1583 (24/24)), and clade 8 (ST10932 and ST13533 (21/21)). A small subset of clade 14 (ST1893 (12/54)) and clade 11 (ST1588 (11/40)) also carried the *tetM* plasmid.

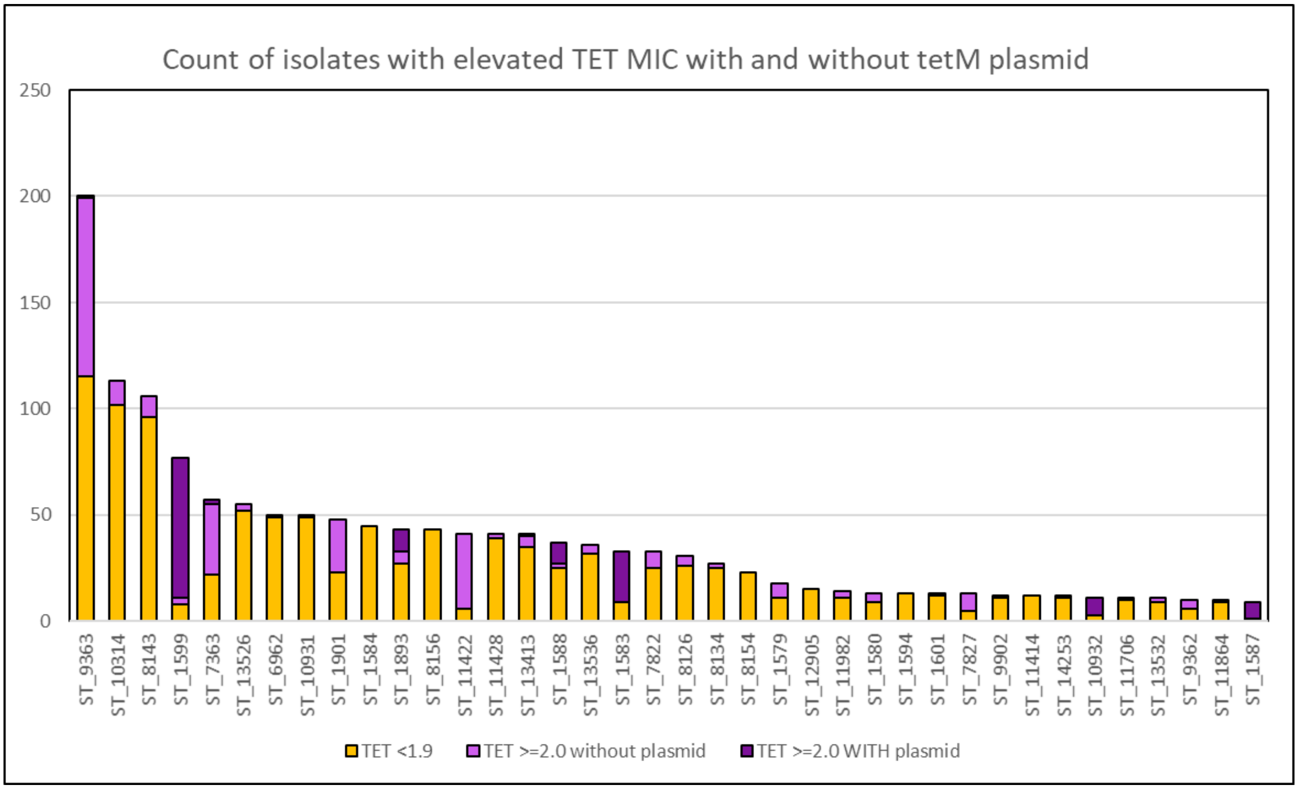

References

1. Wood DE, Salzberg SL. Kraken: ultrafast metagenomic sequence classification using exact alignments. Genome Biol. 2014;15(3):R46.
2. Gupta A, Jordan IK, Rishishwar L. stringMLST: a fast k-mer based tool for multilocus sequence typing. Bioinformatics. 2017;33(1):119-21.
3. Martin M. Cutadapt removes adapter sequences from high-throughput sequencing reads. EMBnetjournal. 2011;17(1):10-2.
4. Bankevich A, Nurk S, Antipov D, Gurevich AA, Dvorkin M, Kulikov AS, et al. SPAdes: a new genome assembly algorithm and its applications to single-cell sequencing. J Comput Biol. 2012;19(5):455-77.
5. Gurevich A, Saveliev V, Vyahhi N, Tesler G. QUAST: quality assessment tool for genome assemblies. Bioinformatics. 2013;29(8):1072-5.
6. Thomas JC, Seby S, Abrams AJ, Cartee J, Lucking S, Vidyaprakash E, et al. Evidence of Recent Genomic Evolution in Gonococcal Strains With Decreased Susceptibility to Cephalosporins or Azithromycin in the United States, 2014-2016. J Infect Dis. 2019;220(2):294-305.
7. Deatherage DE, Barrick JE. Identification of mutations in laboratory-evolved microbes from next-generation sequencing data using breseq. Methods Mol Biol. 2014;1151:165-88.
8. Li H, Durbin R. Fast and accurate short read alignment with Burrows-Wheeler transform. Bioinformatics. 2009;25(14):1754-60.
9. Li H, Handsaker B, Wysoker A, Fennell T, Ruan J, Homer N, et al. The Sequence Alignment/Map format and SAMtools. Bioinformatics. 2009;25(16):2078-9.
10. Kwong JC, Goncalves da Silva A, Dyet K, Williamson DA, Stinear TP, Howden BP, et al. NGMASTER:in silico multi-antigen sequence typing for Neisseria gonorrhoeae. Microb Genom. 2016;2(8):e000076.
11. Demczuk W, Sidhu S, Unemo M, Whiley DM, Allen VG, Dillon JR, et al. Neisseria gonorrhoeae Sequence Typing for Antimicrobial Resistance, a Novel Antimicrobial Resistance Multilocus Typing Scheme for Tracking Global Dissemination of N. gonorrhoeae Strains. J Clin Microbiol. 2017;55(5):1454-68.
12. Treangen TJ, Ondov BD, Koren S, Phillippy AM. The Harvest suite for rapid core-genome alignment and visualization of thousands of intraspecific microbial genomes. Genome Biol. 2014;15(11):524.
13. Croucher NJ, Page AJ, Connor TR, Delaney AJ, Keane JA, Bentley SD, et al. Rapid phylogenetic analysis of large samples of recombinant bacterial whole genome sequences using Gubbins. Nucleic Acids Res. 2015;43(3):e15.
14. Stamatakis A. RAxML version 8: a tool for phylogenetic analysis and post-analysis of large phylogenies. Bioinformatics. 2014;30(9):1312-3.
15. Tonkin-Hill G, Lees JA, Bentley SD, Frost SDW, Corander J. Fast hierarchical Bayesian analysis of population structure. Nucleic Acids Res. 2019;47(11):5539-49.
16. Letunic I, Bork P. Interactive Tree Of Life (iTOL) v5: an online tool for phylogenetic tree display and annotation. Nucleic Acids Res. 2021;49(W1):W293-W6.
17. Yu G, Smith, D.K., Zhu, H., Guan, Y., Tsan-Yuk Lam, T. GGTREE: an R package for visualization and annotation of phylogenetic trees with their covariates and other associated data. Methods in Ecology and Evolution. 2017;8:28-36.
18. Ma KC, Mortimer TD, Hicks AL, Wheeler NE, Sanchez-Buso L, Golparian D, et al. Adaptation to the cervical environment is associated with increased antibiotic susceptibility in Neisseria gonorrhoeae. Nat Commun. 2020;11(1):4126.
19. Katoh K, Standley DM. MAFFT multiple sequence alignment software version 7: improvements in performance and usability. Mol Biol Evol. 2013;30(4):772-80.
